# Rising surface pressure over Tibetan Plateau strengthens indian summer monsoon rainfall over northwestern India

**DOI:** 10.1038/s41598-022-12523-8

**Published:** 2022-05-21

**Authors:** Randhir Singh, Neeru Jaiswal, C. M. Kishtawal

**Affiliations:** Space Applications Centre, Indian Space Research Organisation (ISRO), Ahmedabad, 380015 India

**Keywords:** Climate sciences, Environmental sciences, Hydrology

## Abstract

The dipole pattern (wetting over northwestern India and drying over the Indo-Gangetic plains and northeast India) in the rainfall trends is reported in many earlier studies. The exact cause of the rainfall trends’ asymmetry remains unclear. We show that increasing trends over the northwestern parts are closely associated with the rise in surface pressure over the Tibetan Plateau. The surface pressure over Tibetan Plateau shows increasing trends (0.23 hPa decade^−1^, p < 0.01) during 1979–2020. Easterlies across northwest India and southerlies over east India show rises of − 0.26 ms^−1^ decade^−1^ and 0.15 ms^−1^ decade^−1^, respectively, in line with Tibetan surface pressure trends. Water vapour transfer across northwest India has increased as a result of these changes in circulation. Increased lower-level easterlies carried more water vapour from the Bay of Bengal over northwest India. At the same time, stronger mid-level southerlies drove extratropical dry air out of India, strengthening the rainfall generating mechanism. Rising easterlies in northwest India also enhance vorticity along the monsoon trough, which promotes rainfall generation. Concurrently, because of the high surface pressure over Tibet, the circulation intensity of the mid-tropospheric cyclone over East India was weakened, resulting in less rain in the Indo-Gangetic region. The present study proposes that an increase in the surface pressure over Tibetan Plateau is an important factor contributing to the dipole pattern in the ISMR trends, particularly upward trends in rainfall over northwest India

## Introduction

The Indian summer monsoon (ISM) is the strongest component of the global monsoon system, bringing 70–90% of India’s yearly rainfall^1^. The amount of ISM rainfall (ISMR) varies considerably from year to year, which significantly impacts India’s agriculture, socio-economic development, disaster management, and hydrological planning^2^. The ISMR pattern has changed as a result of climate variability and climate change, in addition to interannual fluctuation. As a result, understanding the underlying factors and examining historical trends in ISMR on various spatial and temporal scales is crucial for planning and policymaking in the highly populated, monsoon-dependent Indian region^3^.

Several studies have examined the interannual variations of the ISMR, indicating strong teleconnections between the ISMR and large-scale climate forcing like the EL-Nino-Southern Oscillation (ENSO)^4–6^, the Indian Ocean Dipole^7–10^, the tropical Atlantic sea surface temperature^11–15^, and the surface temperature over the Middle-East^16^. The nature of these teleconnections and the physical mechanisms of their interaction are still being explored.

Similarly, several studies have looked into long-term ISMR trends. These trends are debated extensively due to differences in the rainfall data sets used in the analysis^17–19^. Most research has found that the ISMR has been trendless over the last four decades, especially on a national scale, with large geographic variability in the trends^.^ The evidence suggests a significant decrease in ISMR over the Indo-Gangetic plains, North East Indian regions, and central-eastern India, along with an increase in rainfall over northwest India^13,14,16,20–25^. At the same time, the frequency and severity of heavy rainfall events in western and central India have increased significantly. Low pressures/depressions occurring in the Arabian Sea and an increase in moisture supply are thought to be the primary causes of increased rainfall activity, particularly intense rainfall events^13,14,20,24^. The increase in rainfall across North-West India is related to a rise in surface temperature over Iran^16^. The higher surface temperature over Iran resulted in a cyclonic circulation with stronger northerlies and westerlies over the northern Arabian Sea, favouring deep convection over North-West India due to adequate moisture availability^16^. The increase in surface temperature over Iran is due to a mid-latitude wave train that propagates from the north-eastern Atlantic and over Europe to Iran^16^.

The effect of urbanization in regulating the frequency of heavy rain events was highlighted^26^. The role of global-scale anthropogenic forcings like GHGs, as well as regional-scale forcings like aerosols and land-use/land-cover changes, has also been examined in previous research for the decrease in rainfall^27,28^. Other factors that may have influenced ISMR variability include El Nino frequency, weakened monsoonal circulation, increased air pollution, and warming of the Indian Ocean^29,30^.

The Tibetan Plateau is the world’s highest and largest Plateau, with a mean altitude of over 4000 m and a surface area of about 3,000,000 km^2^. The Tibetan Plateau has a significant impact on local weather and atmospheric circulation because it acts as a geographical barrier and elevated heat source^31–33^.The strength of following ISMR is highly connected with the springtime temperature over the Tibetan Plateau^34^. Other study suggests that the Tibetan Plateau influences the ISMR mostly by blocking cool, dry air from northwest Tibet, with heating playing a minor contribution^35^. Furthermore, while Tibetan heating (as measured by moist static energy of surface air) is significantly correlated with early (20 May to 15 June) and late (1 September to 15 October) monsoon season rainfall, the correlation between Tibetan heating and rain in the main monsoon period (15 June to 31 August) is insignificant^36^. It has also been demonstrated that Tibetan heating is mostly independent of ENSO, and that both (i.e. Tibetan heating and ENSO) may explain a large portion of early and late-season rainfall variability^36^. According to a recent study, the Tibetan Plateau summer monsoon and monsoonal rainfall over northern India have an inverse relationship^37^.

The present study proposes that the rise in the surface pressure above the Tibetan Plateau as a result of a warmer climate is an important factor contributing to the upward rainfall trends in northwest India and, to a lesser extent, downward rainfall trends in the Indo-Gangetic Plains and northeast India. This aspect has not been addressed in any earlier work, to the best of the authors’ knowledge.

## Results

### Rainfall trends

**Figure 1:**
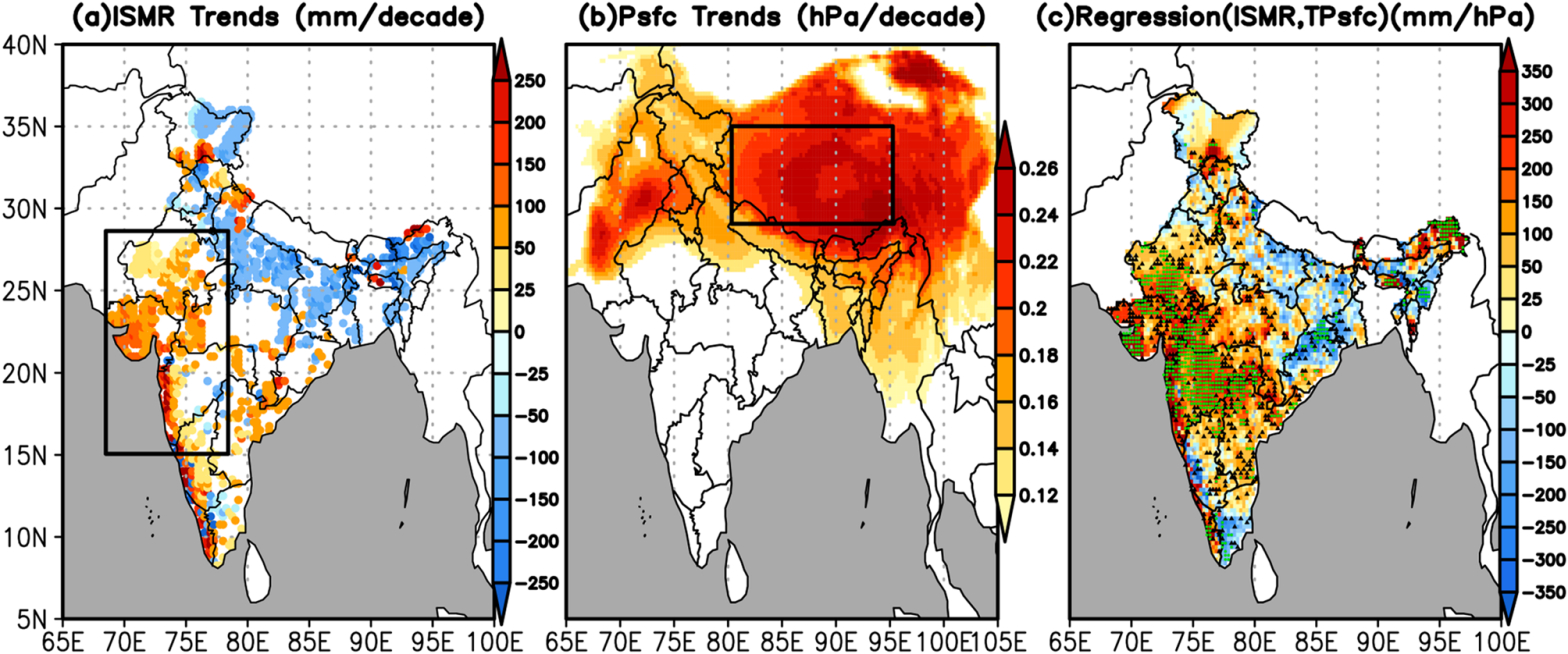
(**a**) linear trends of rainfall (mm/decade), (**b**) linear trends of surface pressure (hPa/decade), and (**c**) regression of the ISMR against the Tibetan surface pressure (TPsfc) (mm hPa^−1^). Only those trends that are significant above 95% confidence level are shown in (**a**) and (**b**). In (**c**), the green color circles show the regions where regression is significant above 95% confidence level and the black color triangles show the regions where regression is significant between 90 and 95% confidence levels. The entire analysis is for June–September, during 1979–2020. The rectangles in (**a**) and (**b**) depict northwest India (15°N–28°N, 68°E–78°E) and the Tibetan region (29°N–35°N, 80°E–95°E), respectively, which were considered in the analysis. The figures are developed using open source software GrADS (Grid Analysis and Display System) version 2.2.1 (http://cola.gmu.edu/grads/downloads.php).

Fig. 1a depicts the spatial distribution of the JJAS (June through September) rainfall trend of the last 42 years (1979–2020). Significant negative trends (50 to 200 mm decade^−1^) were observed over the Indo-Gangetic plains (IGP, states like Uttar Pradesh, Bihar, Jharkhand, North West Bengal) and northeast India (states like Arunachal Pradesh, Assam and Meghalaya), with some isolated pockets over states like Haryana, Punjab, North West Himachal Pradesh and Jammu-Kashmir, please refer to Fig. S1 (Supplementary) for locating the Indian states. Significant positive trends (50 to 200 mm decade^−1^) were observed over northwest India (states like Rajasthan, Gujarat and Western Madhya Pradesh), West Central and Peninsular India (Viz Coastal Maharashtra, Karnataka and Kerala), and northeast Andhra Pradesh. Significant positive trends were also observed over isolated regions of states like Uttarakhand, Himachal Pradesh, South Jammu-Kashmir and North East Arunachal Pradesh.

Several studies have discussed the increasing ISMR over the northwest parts of India and in the Western Ghats and decreasing ISMR along the Himalayan foothills and northeast India*.* Most of the earlier studies emphasized the role of enhanced evaporation (in the background of global warming) and moisture advection over the Arabian Sea in increasing rainfall activities over northwest India and the Western Ghats^16,20,24^. The land use land cover change has been shown to have a significant role in the decreasing trends in ISMR over the Himalaya foothills and northeast India^27,38,39^.

A recent study proposed that the dipole structure in rainfall trends is closely connected with the Azores high^40^. The Azores High is accompanied by strong subsidence that causes widespread upper tropospheric convergence. The Rossby wave source is enhanced across the North Atlantic as a result of the magnified convergence, which intensifies the circum-global rotating Rossby wave train and geopotential height (GPH) anomalies over the Eurasian region. The Eurasian Rossby wave cascades down, causing negative, positive, and negative upper tropospheric GPH anomalies over the north Mediterranean, northwest India, and northeast India, respectively. This dipole GPH anomaly over north India causes the Tibetan high to migrate westward, causing the heavy convection zone to shift from northeast India to the west and central India, resulting in a west-east dipole rainfall pattern. The active phase of the Azores high has a large influence on the ISMR than the weak phase, and hence the overall connection between the two is not that strong^40^. By computing the correlation between surface pressure averaged over the Azores High (AH, 25°N–45°N, 300°E–345°E) and ISMR over northwest India, we were able to validate the poor relationship between the Azores High and ISMR. Although there is a positive correlation between the two (r = 0.201), it is insignificant. Furthermore, as previously stated, rainfall in northwest India is increasing, while average surface pressure in the Azores High is decreasing (− 0.102 hPa/decade), at 85% confidence level. This verifies the Azores High’s poor overall relationship to the ISMR over northwest India. On the other hand, the relationship between surface pressure over the Tibetan Plateau and ISMR over northwest India is much stronger and significant, as detailed later.

As a result, this study proposes that the rise in surface pressure over the Tibetan plateau, which is occurring in the context of global warming, is a crucial factor contributing to the higher trend in rainfall in India’s northwest. We did this by looking at the trend of surface pressure over the Tibet plateau, as well as other aspects of global monsoon circulation that are detailed later in the paper.

### Regressions between Tibetan surface pressure and ISMR

Fig. 1b shows the spatial distribution of the trends in the JJAS surface pressure for 1979–2020. A significant (p < 0.05) increase in the surface pressure is noticed, mainly over the Tibetan Plateau, with the most prominent increasing trends over the high mountain areas. For further analysis, we defined an area (29°N–35°N, 80°E–95Eº) with the largest trends in surface pressure. This area-averaged surface pressure over the Tibetan Plateau is referred to hereafter as TPsfc. There is a significant increase in TPsfc (0.23 hPa decade^−1^, p < 0.01) (Supplementary, Fig. S2a). Surface pressure trends over the Tibetan Plateau are consistent with the trends documented in earlier studies^41–43^, which analyzed both in-situ and model reanalyses data.

Further, to study the possible connection between asymmetry in the rainfall trends and large scale atmospheric circulation, the regional average of JJAS rainfall is defined over the northwest region (15°N–28ºN, 68°E–78ºE hereafter NWIR). The NWIR displays an increasing trend of 32.20 mm decade^−1^ (p < 0.05) during 1979–2020 (Supplementary, Fig. S2b). The year-to-year variations of detrended and standardized NWIR and TPsfc are significantly positively correlated (r = 0.48, p < 0.01) (Supplementary, Fig. S2c). The regression of TPsfc with ISMR at each grid over the Indian region is computed to understand the spatial pattern of the association between TPsfc and ISMR (Fig. 1c). It is interesting that spatial variation of regressions is very similar to the rainfall trends (Fig. 1a); positive regression over northwest India, west coast of southern India, and negative regression towards IGP region and northeast India. This confirms a close association between the surface pressure over the Tibetan Plateau and rainfall trends over the Indian region. The Tibetan Plateau controls rainfall variability more over the northwestern parts of India, as the patterns of regression are contiguously large and statistically significant. Despite the uneven and scattered regression pattern, there is a significant (p < 0.1) out-of-phase relationship between ISMR and TPsfc over IGP and the northeast region.

ENSO, North Atlantic Oscillation (NAO), Arctic Oscillation (AO), Pacific Decadal Oscillation (PDO), and North Pacific Pattern (NP) are some of the major teleconnection patterns that influence the variability of Indian summer monsoon rainfall^4–6,11–15,44–48^. Correlations of the above-mentioned climate drivers with ISMR (i.e. NWIR) and surface pressure (TPsfc) are computed to examine whether ISMR and TPsfc are independent of the relation of ISMR with various measures of tropical and extratropics climate variability. The correlation of the JJAS Nino3.4 index with NWIR is − 0.34 (p < 0.05). The relationship between TPsfc and Nino3.4 is weak and non-significant; it seems that ENSO has no role in TPsfc and NWIR association. PDO correlation with TPsfc is 0.32 (p < 0.05), whereas the correlation between NWIR and PDO is insignificant. Though a significant positive correlation between TPsfc and NP exists, the correlation of NP with NWIR is weak and non-significant. The correlations r(NWIR/AO), r(NWIR/NAO), r(TPsfc/AO), and r(TPsfc/NAO) are also non-significant. It’s worth mentioning that earlier studies have found a strong link between ISMR and the North Pacific Pattern (NP) and the Pacific Decadal Oscillation (PDO)^46–49^. However, there was no significant association between rainfall in northwest India and NP or PDO. The correlation of NP and PDO with ISMR at each grid over the Indian region, on the other hand, shows that NP is considerably positively connected with rainfall over states such as Uttar Pradesh, scattered pockets over eastern Rajasthan, and peninsular India (states like Andhra Pradesh and southern Maharashtra). PDO, on the other hand, has a substantial negative correlation with rainfall in the IGP region and southern India (states such as Karnataka and Tamilnadu), but a large positive correlation in the eastern states. Overall, the results show that the relationship between NWIR and surface pressure across the Tibetan plateau is unaffected by main climate variability drivers, which is in line with previous findings^36^.

### The linkage between ISMR and Tibetan surface pressure

To understand the physical mechanism that connects the Tibetan surface pressure and ISMR, regression and composites analysis are used to examine atmospheric circulation changes associated with TPsfc variability.

### The connection between TPsfc and 550 hPa circulation pattern

**Figure 2:**
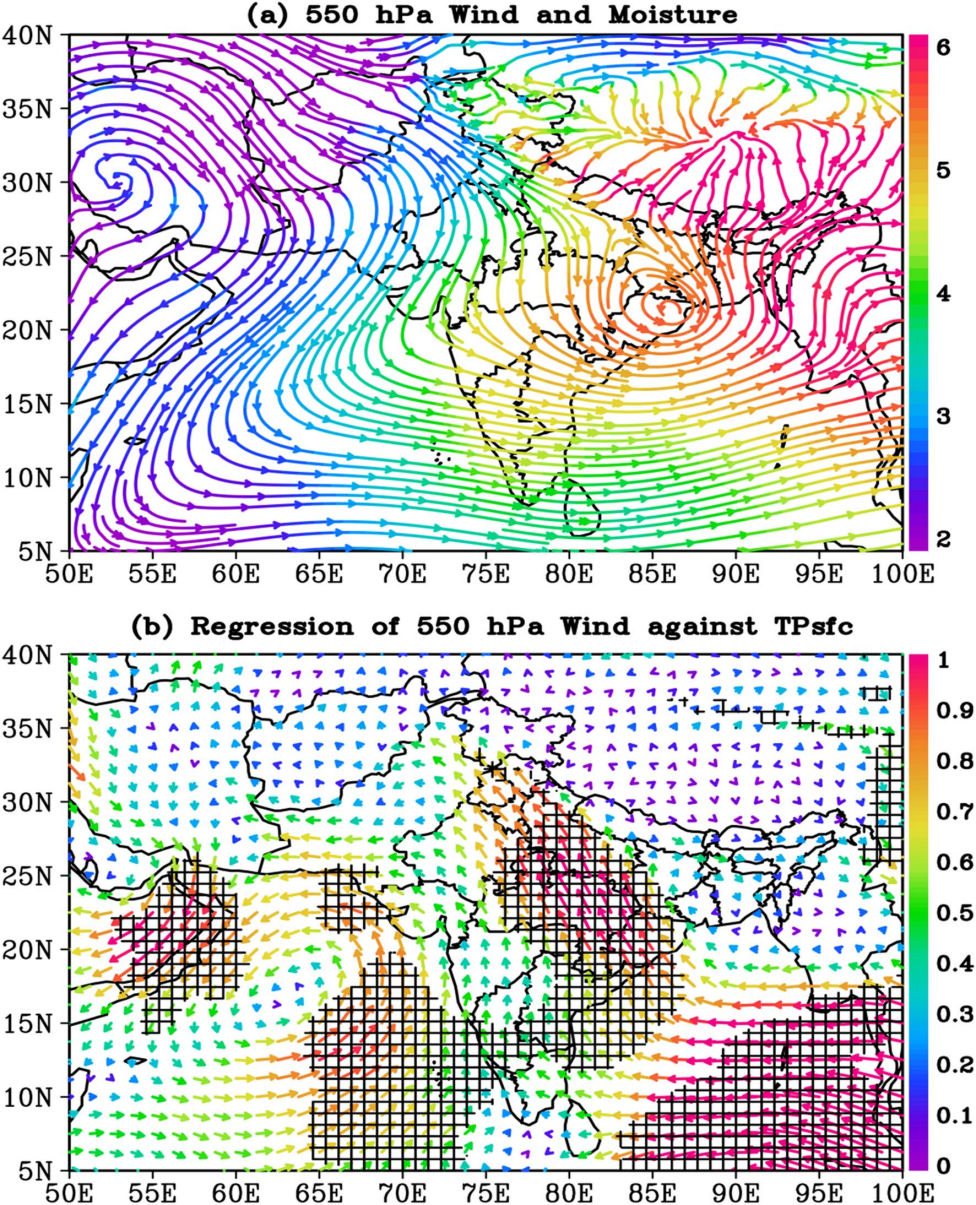
(**a**) Climatology (JJAS, 1979–2020) of 550 hPa wind circulation (streamlines) and moisture (i.e. specific humidity, g kg^−1^) color, and (**b**) the regression of 550 hPa wind against TPsfc (ms^−1^ hPa^−1^). The black hatch area in (**b**) indicates the regions where regressions are significant above 95% confidence level. The entire analysis is for June–September, during 1979–2020. The figures are developed using open source software GrADS (Grid Analysis and Display System) version 2.2.1 (http://cola.gmu.edu/grads/downloads.php).

Because the average surface pressure in the Tibetan region (29°N–35°N, 80°E–95°E) is 565 hPa, the TPsfc’s maximum impact on circulation is anticipated to be around 550 hPa. For the period 1979–2020, regression analysis between JJAS 550 hPa wind and TPsfc time series is performed. Figure 2 shows the long-term mean 550 hPa wind and moisture, as well as regression between 550 hPa wind and TPsfc. The 550 hPa circulation is characterized by high pressure (anticyclonic circulation) above Iran and low pressure (cyclonic circulation) above East India (northwest of Bay of Bengal, i.e. the states of Bihar, Jharkhand, Orissa, and West Bengal) based on long-term mean circulation patterns (Fig. 2a). The anticyclonic circulation over Iran is formed by summertime sinking over subtropical desert regions^50^, whereas the cyclonic circulation over the northwestern part of the Bay of Bengal is a part of quasi-stationary mid-tropospheric cyclones^51^. Most of India is covered by north-westerly flow as a result of these high and low-pressure systems. These north-westerly winds bring dry air from the extratropical zone to India, particularly in the northwestern part of the country. Over the Tibetan Plateau, winds from the eastern flank of the East India low and the north-eastern flank of the Iranian high are converging. Over the Arabian Sea, a well-defined trough can also be seen spreading from the northeast to the southwest (Fig. 2a).

According to the regression analysis, significant south easterly anomalies are seen over central-east India, north India, and the eastern Bay of Bengal (Fig. 2b). A cyclonic circulation anomaly has been observed in the north-central Arabian Sea. This implies that an increase (decrease) in Tibetan surface pressure corresponds to significant south easterly (north-westerly) anomaly over India and a cyclonic (anticyclonic) circulation anomaly over the north-central Arabian Sea. Extra tropical winds from high pressure over Iran converge from two directions over the Tibetan plateau (as seen in Fig. 2a). One branch flows over northern India before reaching Tibetan Plateau. At the same time, the other branch covers north India, central India, and the north section of the Arabian Sea before heading to the low over east India and finally converging over the Tibetan Plateau. As a result, increased Tibetan surface pressure will weaken the low-pressure system over east India, limiting north-westerly winds over the country. A significant positive relationship (r = 0.79, P < 0.01) between TPsfc and 550 hPa geopotential height over East India (22°N–30°N, 78°E–86°E) further confirms the weakening of the low. The dry influx over the country from extratropical regions will be limited by the reduced intensity of north-westerly winds. The regression of TPsfc with 550 hPa zonal and meridional wind components is shown separately in Fig. S3. Except for a substantial association across the southeastern Bay of Bengal, where values range from − 0.5 to − 1.4 ms^−1^ hPa^−1^ (p < 0.05), the regression magnitude reveals that 550 zonal winds are not significantly associated with TPsfc over the Indian region (Fig. S3a). The regression between TPsfc and 550 hPa meridional winds, on the other hand, is quite strong and extends over a considerably greater area, particularly in the western Arabian Sea and the region extending from the east coast to the IGP, with regression values ranging from 0.2 to 1.2 ms^−1^ hPa^−1^ (p < 0.05) (Fig. S3b).

Figure S4 illustrates the interannual variability of area-averaged (15°N–28°N, 75°E–85°E) JJAS 550 hPa meridional wind (hereafter V550), TPsfc, and NWIR to better understand the relationship between surface pressure over the Tibetan Plateau and NWIR. The area utilized to average the winds is determined by the most significant values of the TPsfc-winds regression, discussed earlier (Fig. S3b). Figure S4a shows that the TPsfc and V550 time series are highly correlated (r = 0.52, p < 0.01). Likewise, the interannual variability of NWIR is strongly associated with V550, with a correlation of 0.74 (p < 0.01) (Fig. S4a). Furthermore, trends in V550 are analyzed, which reveals a strong connection between increased TPsfc and weakening northerly (0.15 ms^−1^decade^−1^, p < 0.01) (Fig. S4b). Enhanced moisture-laden south easterlies from the Bay of Bengal (i.e. weak extra tropical north westerlies, Figs. S3b, S4b) would increase moisture content and subsequently rainfall activity over northwestern India during high TPsfc. The regression of 550 hPa specific humidity with V550 backs up the above arguments (Fig. S5). Across the Arabian Sea and the northern central parts of India, V550 is significantly positively connected to 550 hPa specific humidity (Fig. S5). Interannual variability between area-averaged (15°N–28°N, 68°E–78°E) 550 hPa specific humidity (hereafter H550) and V550 is highly correlated (r = 0.78, p < 0.01, Fig. S6a). Further, H550 displays significant positive trends (0.15 g kg^−1^decade^−1^, p < 0.01, Fig. S6b), which are consistent with trends in V550 (Fig. S4b). Additionally, cyclonic circulation prevails over the north-central Arabian Sea, with its southeastern flank capable of transporting moisture from the Arabian Sea to northwest India, as previously stated. The importance of cyclonic circulation over the northern Arabian Sea in carrying moisture over northwest India is also emphasized in earlier study^16^.

Furthermore, based on the interannual variability of TPsfc, ten years with the highest TPsfc (hereinafter HTPsfc) and ten years with the lowest TPsfc (hereinafter LTPsfc) are chosen to construct the circulation composites. The circulation anomaly patterns obtained using composite differences (not shown for brevity) are quite similar to those found using regression analysis, revealing a substantial relationship between TPsfc and circulation anomalies in India.

### The connection between TPsfc and 850 hPa circulation pattern

**Figure 3:**
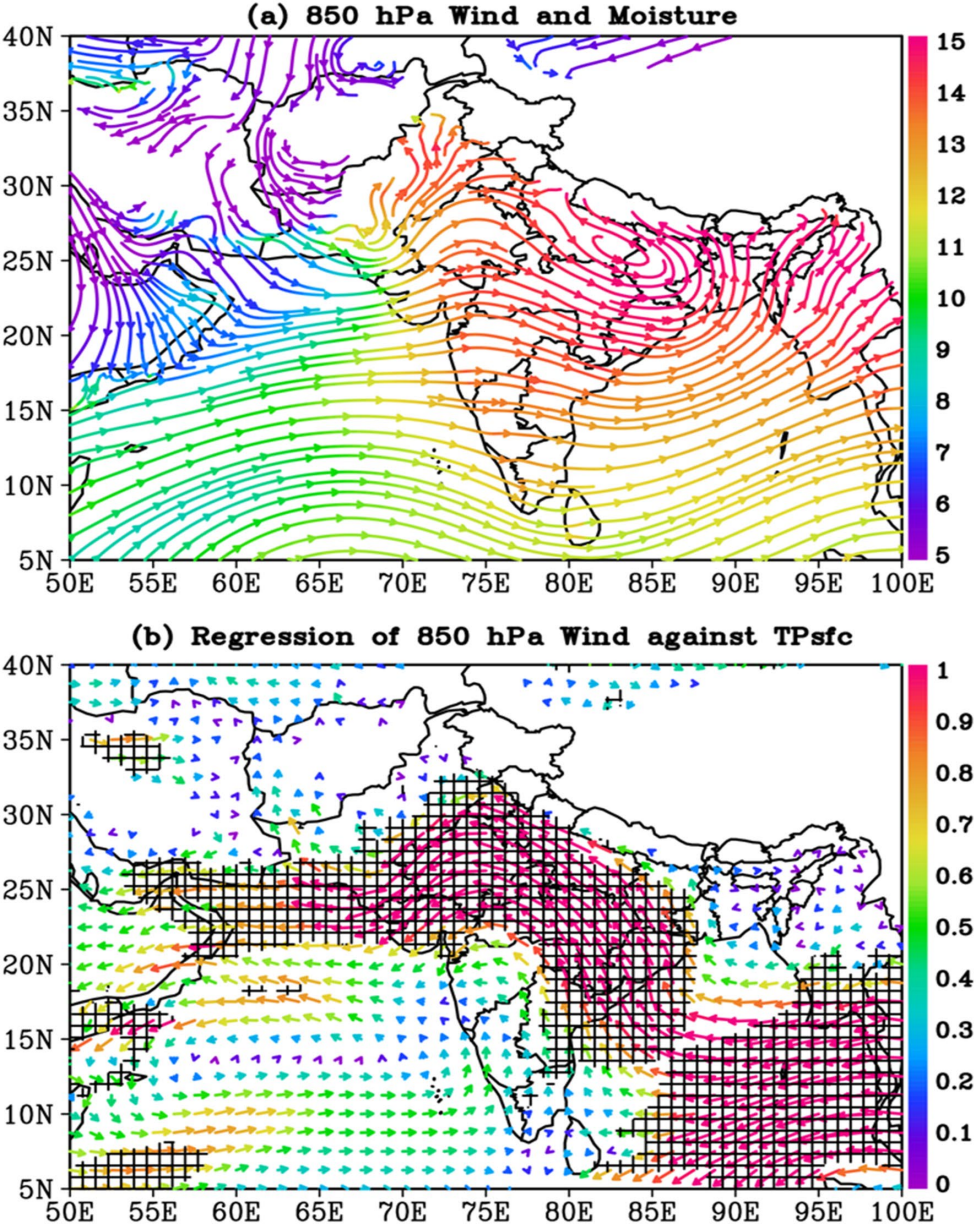
(**a**) Climatology (JJAS, 1979–2020) of 850 hPa wind circulation (streamlines) and moisture (i.e. specific humidity, g kg^−1^) color, and (**b**) the regression of 850 hPa wind against TPsfc (ms^−1^ hPa^−1^). The black hatch area in (**b**) indicates the regions where regressions are significant above 95% confidence level. The entire analysis is for June–September, during 1979–2020. The figures are developed using open source software GrADS (Grid Analysis and Display System) version 2.2.1 (http://cola.gmu.edu/grads/downloads.php).

A notable feature of the lower level wind is the enhanced westerly flow across most of India, with a large cyclonic circulation following the monsoon trough evident across the IGP region (Fig. 3a). The monsoon flow over India at 850 hPa is also much stronger than the mid-tropospheric flow (550 hPa) (Fig. 3a). The JJAS 850 hPa wind and TPsfc time series also have a strong connection, as per the regression analysis (Fig. 3b). In fact, the association between the TPsfc and 850 hPa winds is much stronger than the association between the TPsfc and 550 hPa winds, particularly the connection between the TPsfc and zonal wind across northwest India. The magnitude of regression ranges from − 0.8 to − 1.6 ms^−1^ hPa^−1^ (p < 0.05, Fig. S7a). The 850 and 550 hPa regression patterns for meridional winds are fairly similar, except that in the case of 850 hPa, the regression is not significant across the western Arabian Sea, and instead, a small but significant negative regression zone emerges across western India (Fig. S7b).

**Figure 4:**
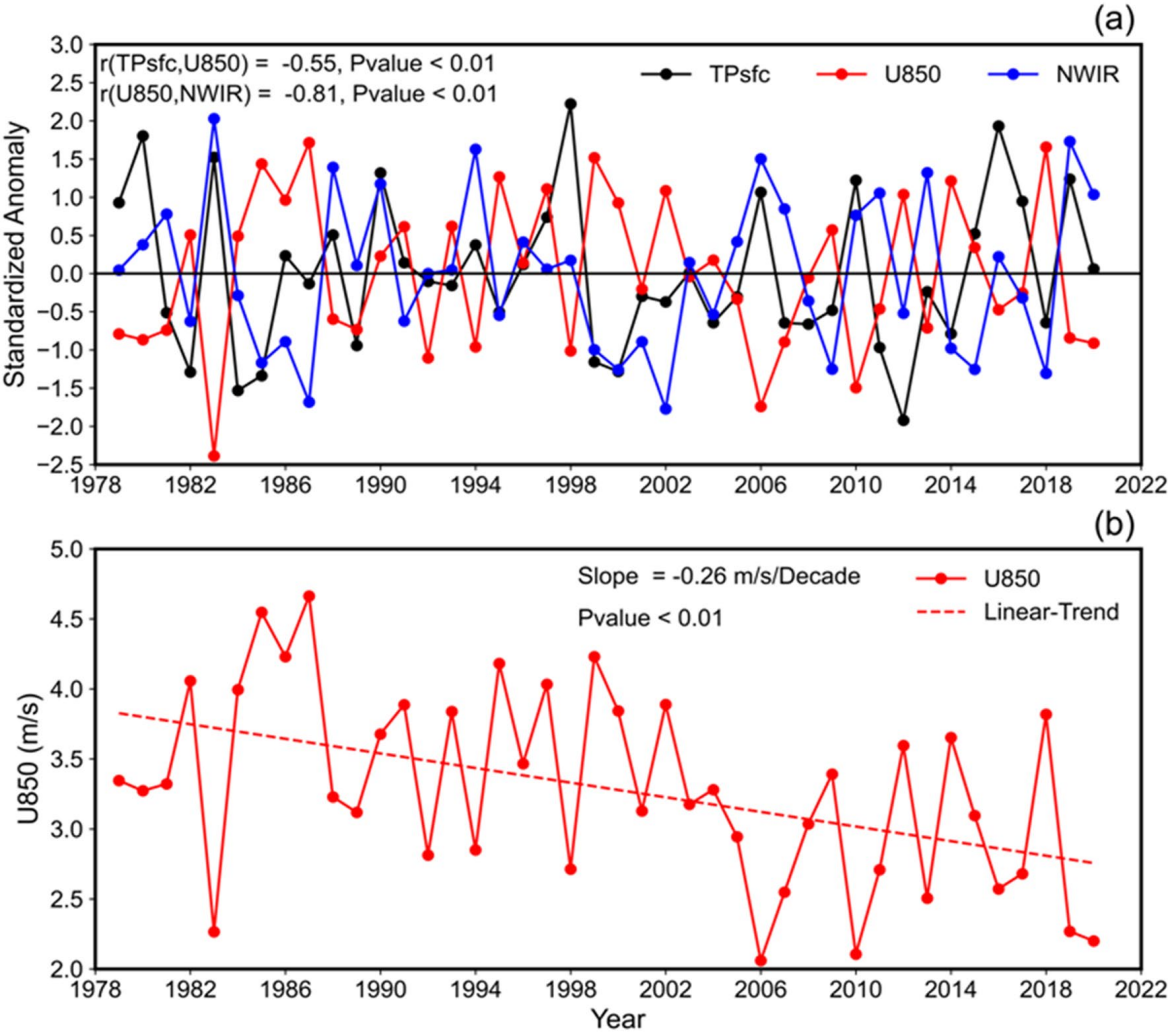
(**a**) Detrended and standardized time series of area averaged surface pressure over the Tibetan Plateau (TPsfc, 29°N–35°N, 80°E–95°E), 850 hPa zonal wind (U850, 20°N–30°N, 68°E–78°E), area averaged rainfall (NWIR, 15°N–28°N, 68°E–78°E), and (**b**) time series of 850 hPa zonal wind (U850, 20°N–30°N, 68°E–78°E). The entire analysis is for June–September, during 1979–2020. The figures are created with Python version 3.9.1 (https://www.python.org), an open source programming language.

To show how zonal wind anomalies over northwestern India can influence the ISMR, the interannual variability of the area-averaged JJAS 850 hPa zonal wind is compared with TPsfc. The strong link between surface pressure over the Tibetan Plateau and zonal wind over northwest India (U850, 22°N–30°N, 68°E–78°E) is reflected in the interannual variability of U850 and TPsfc. A high negative correlation exists between the two time series (r = − 0.55, p < 0.01, Fig. 4a). Similarly, with a − 0.81 (p < 0.01, Fig. 4a) correlation, NWIR interannual variability is strongly linked to U850. In addition, U850 trends are examined, revealing a strong relationship between rising TPsfc and rising easterlies (− 0.26 ms^−1^decade^−1^, p < 0.01, Fig. 4b). So, due to an increase in surface pressure over the Tibetan plateau, northwestern India is experiencing easterlies. The question now is how easterlies across northwest India increase rainfall. Increased easterlies could transport more moisture from the Bay of Bengal to the northwestern part of India, leading to increased rainfall activity. Over the northwestern part of India, the 850 hPa zonal wind and specific humidity (H850, 15°N–28°N, 68°E–78°E) are found to be negatively correlated (r = − 0.63, p < 0.01, Fig. S8a). H850 trends are also examined, and they are significantly positive (0.19 g kg^−1^decade^−1^, p < 0.01, Fig. S8b), reflecting the U850 trends.

Further, during the summer monsoon season, lows and depressions are key rain-producing synoptic-scale disturbances. These lows/depressions originate over the Bay of Bengal, as well as the South China Sea on occasion, and migrate west-northwest along the monsoon trough (an elongated low-pressure zone running parallel to the Himalayan Mountains in a west to east direction from Northwest Rajasthan to the Bay of Bengal^52,53^. These systems have a 4 to 5-day life cycle and are characterized by low-pressure zones and counter-clockwise winds. Because the monsoon trough is characterized by easterlies in the north and westerlies in the south, stronger easterlies will increase the vorticity in the trough, resulting in increased rainfall. Interannual variability of area-averaged zonal wind (i.e. U850) and vorticity (Z850, 15°N–28°N, 70°E–78°E) is depicted in Fig. S8a. Increased easterlies enhance vorticity, as both time series are significantly negatively correlated (r = − 0.66, p < 0.01, Fig. S8a). Earlier study also stated that active monsoon conditions are supported by easterlies across northern India and a broad 500 hPa high over the Tibetan Plateau^54^.

**Figure 5:**
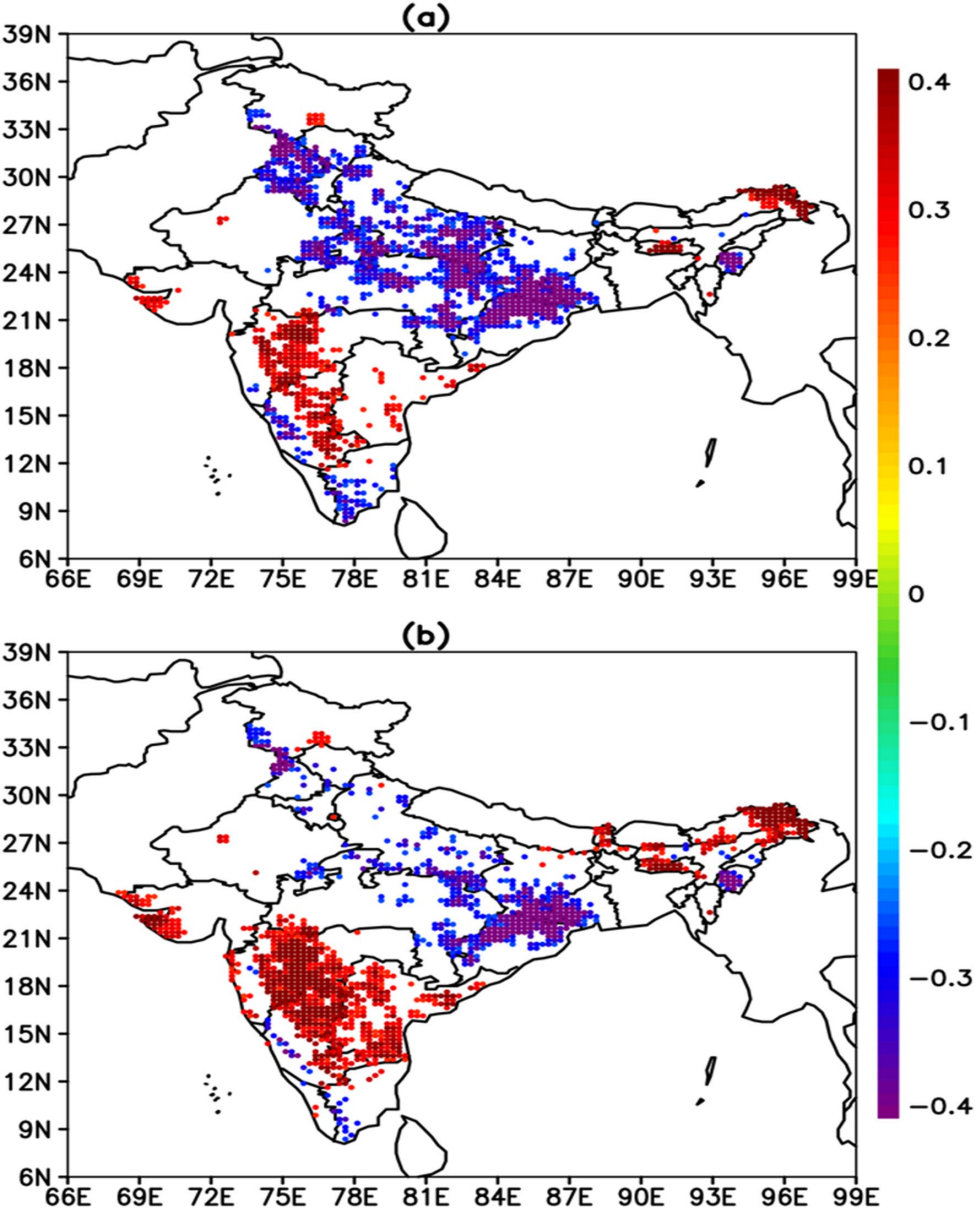
Correlation between area-averaged (22°N–30°N, 78°E–86°E) geopotential height [(**a**) for 850 hPa and (**b**) for 550 hPa] and ISMR at each grid point over India. Only correlations that are statistically significant above 90% confidence interval are displayed. A value of |r| larger than 0.31 is significant above the 95% confidence level. The entire analysis is for June–September, during 1979–2020. The figures are developed using open source software GrADS (Grid Analysis and Display System) version 2.2.1 (http://cola.gmu.edu/grads/downloads.php).

The circulation anomalies linked to TPsfc variability, as previously mentioned, clearly explain the underlying mechanism by which TPsfc regulates rainfall over northwest India. However, as earlier mentioned, TPsfc is negatively associated with rain in the IGP region, though the relationship is not as strong as in northwest India. The question now is how the TPsfc is influencing rainfall over the IGP region. To provide an explanation, we calculated the correlation between area-averaged (22°N–30°N, 78°E–86°E) geopotential height (both 850 hPa and 550 hPa) and ISMR rainfall at each grid point over India. Based on the correlation between TPsfc and geopotential height, the region used to obtain area-averaged geopotential height was identified. In the IGP region, both 850 and 550 hPa area-averaged geopotential heights are significantly inversely associated to rainfall (Fig. 5). In addition, the 850 hPa geopotential height has a stronger link than the 550 hPa geopotential height. Because the eastern flank of the low pressure over East India (i.e. the northwest Bay of Bengal) is converging over the Tibetan plateau, the high surface pressure over Tibet reduces the circulation intensity in the low pressure over East India (as shown in Figs. 2a, 3a), resulting in less rain in the IGP region.

### How TPsfc does influences the circulation at the lower levels?

Here, it may be argued how changes in surface pressure at such a high altitude as the Tibetan plateau might affect lower-level circulation. This could be because the atmospheric layer does not behave in isolation; there are significant interactions between winds at various levels. The correlation between 850 hPa zonal wind and 700, 600, 550, and 500 hPa zonal winds in the northwestern part of India (where TPsfc regulates the winds) is 0.85, 0.66, 0.54, and 0.42, respectively, and is significant at 0.01 level, according to our analysis. If there are changes at 550 hPa, there would undoubtedly be changes at 850 hPa as well. Changes in Tibetan surface pressure can also affect the low-level circulation by regulating the regional Hadley circulation, which has ascending and descending branches over north India and the equatorial region, respectively^55,56^. To further explain the high correlation between TPsfc and 850 hPa winds, we computed the correlation of both 850 and 550 hPa winds with TPsfc using daily averaged data from 1979 to 2020. Figure S9 depicts the regional distribution of these correlations, revealing that the pattern of association between TPsfc and winds computed on daily (Fig. S9) and monthly (Figs. 3 and S7) time scales are comparable, especially in northwestern India, East India, and along the IGP region. Furthermore, the association between TPsfc and 550 hPa winds is stronger on a daily time scale than the relationship between TPsfc and 850 hPa winds. However, the association between TPsfc and 850 hPa winds is much greater on a monthly time scale than the relationship between TPsfc and 550 hPa winds. This could be due to the synoptic-scale interaction of lower and middle-level processes. Another reason for the greater impact of TPsfc at lower levels could be because the JJAS 850 hPa circulation is stronger on average across India than the 550 hPa circulation^57^. Also, note that the strong association between TPsfc and zonal winds over the eastern Bay of Bengal that was observed on the monthly (Fig. S7) time scale is not observed on the daily time scale, implying that the strong association between TPsfc and zonal winds over the eastern Bay of Bengal may be the result of synoptic-scale interactions rather than being influenced directly by TPsfc.

### The causal effect of Tibetan surface pressure on ISMR

Though correlation and composite analysis are widely used to understand the relationship between two or more climate variables, it is prone to over-predict the significant relationship when variables have considerable memory (e.g., autocorrelation)^58^. Recently, causality analysis has become one of the most widely used methods in analyzing and establishing the physical links between climate variables^59,60^. Such algorithms can be used to determine if hypothesized relationships are likely to represent real physical processes or are instead artefacts caused by spurious correlations.

Therefore, to understand the co-variability and possible directional causality between Tibetan surface pressure (TPsfc, 29–35ºN, 80–95ºE) and summer rainfall over northwest India (NWIR, 15–28ºN, 68–78ºE) India, 0–15 days lagged correlation coefficient between daily TPsfc and NWIR were calculated, for 1979–2020. Statistical significance was assessed using the Student-test against the null hypothesis of no correlation. For lag 15, which has the lowest number of samples (4494), |r| > 0.03 is significant at a 95% confidence level. TPsfc and NWRI show significant positive correlations for lag 0 to 13 days, with TPsfc leading the NWRI (Supplementary Fig. S10). We also studied the causal relationship between TPsfc and NWIR at different lags. The results suggest a statistically significant causal connection between TPsfc and NWIR, especially for lags 0–13 days (Table 1). The above analysis indicates that surface pressure over Tibetan Plateau leads to rainfall over northwest parts of India and can provide crucial predictive information about rainfall variability over this part of the county.

**Table 1:** Results of Granger Causality Test: area averaged (29°N–35°N, 80°E–95°E) Tibetan surface pressure (TPsfc) to area averaged (15°N–28°N, 68°E–78°E) rainfall, during JJAS (1979–2020).

TPsfc leads rainfall (days)	Numerator degrees of freedom	Denominator degrees of freedom	F-ratio	P-value
1	1	5079	37.73	0.00001
2	1	5037	65.32	0.00001
3	1	4995	66.11	0.00001
4	1	4953	58.35	0.00001
5	1	4911	56.21	0.00001
6	1	4869	53.07	0.00001
7	1	4827	40.33	0.00001
8	1	4785	23.25	0.00001
9	1	4743	13.92	0.0001
10	1	4701	10.67	0.001
11	1	4659	08.60	0.003
12	1	4617	08.35	0.004
13	1	4575	06.28	0.01
14	1	4533	02.38	0.12
15	1	4491	00.57	0.45

### Why is the surface pressure over the Tibetan plateau increasing?

Now the fundamental question arises why is surface pressure increasing over the Tibetan Plateau? The enhanced descending motions in the atmosphere, the increase in atmospheric water vapour loading, and injection of various chemical and by-products of burning fossil fuels (e.g., aerosol and CO_2_ etc.) could be the possible reasons for the rise in the atmospheric mass and hence surface pressure. The net change in the surface pressure due to increased chemical mass (e.g., CO_2_, aerosols, etc.) is likely to be less than 0.01 hPa^61^. However, water vapour loading could be significant in the upward trends of the surface pressure. Therefore, we analyzed water vapour pressure trends over the Tibetan Plateau and found that water vapour pressure has upward trends (0.032 hPa decade^−1^, p < 0.01) for 1979–2020 (Supplementary Fig. S11a). An analysis shows that about ~ 14% increase in the surface pressure over Tibetan Plateau is due to the rise in the water vapour loading. The upward trends in water vapour over the Tibetan plateau are consistent with recently documented findings^62,63^.

We have also analyzed trends in the vertical velocity (i.e. omega) for 1979–2020. Analysis indicates (Supplementary Fig. S11b) that 300 hPa vertical velocities have increasing trends over the Tibetan Plateau, − 0.24 cm hPa^−1^decade^−1^ (p < 0.01), meaning enhanced convective activity, probably due to an increase in the water vapour loading. Note that negative values of vertical velocity correspond to upward motion. Earlier studies have also noticed enhanced convective activities over the Tibetan Plateau^64^. Therefore, the upward trends in the vertical velocity and enhanced convective activities eliminate the possibilities of increased surface pressure due to subsidence.

The uneven warming over Tibetan Plateau could also be one of the reasons for the increase in the surface pressure. Recent studies explained the increases in the surface pressure over the Mongolian Plateau in terms of the rise in the surface temperature around lake Baikal^65,66^. Another study proposed that the rise in the surface pressure over Mongolia is due to the weakening in the extratropical cyclones activity, related to the decrease in the vertical wind shear and atmospheric baroclinicity via uneven surface warming^67^. We computed the correlation between detrended and standardized time series of area-averaged surface pressure and temperature over Mongolia and found a significant association (r = 0.45, p < 0.01) between the two. The temperature trends are robust, 0.44 K decade^−1^ (p < 0.01), which are consistent with pressure trends. The significant positive association between surface pressure and temperature with strong surface warming trends suggests a definite role of local warming in increasing the surface pressure over the Mongolian region. However, unlike the Mongolian area, fragile and non-significant positive correlations (r = 0.14) are observed between area-averaged surface pressure and temperature over the Tibetan Plateau, suggesting the role of some regional/remote forcings in increasing the surface pressure over the Tibetan Plateau.

Literature suggests that surface pressure over a high elevation region like the Tibetan Plateau in a hydrostatic atmosphere contains the signature of the warming below a plateau’s height^41,42,68,69^. Further, the magnitude of increase in the pressure from a given change in the surface temperature increases with altitude^68^. Observational evidence indicates that for a stably stratified atmosphere, the sensitivity of pressure change to surface temperature, for the expected heights of the Tibetan Plateau (3000–4000 m), is of the order of 0.8 to 1 hPa °C^−1^^41,69^. Furthermore, both model reanalysis and in-situ data showed a significant and positive correlation between the surface pressure over the Tibetan Plateau and regional and hemispheric temperature fields^41^.

**Figure 6:**
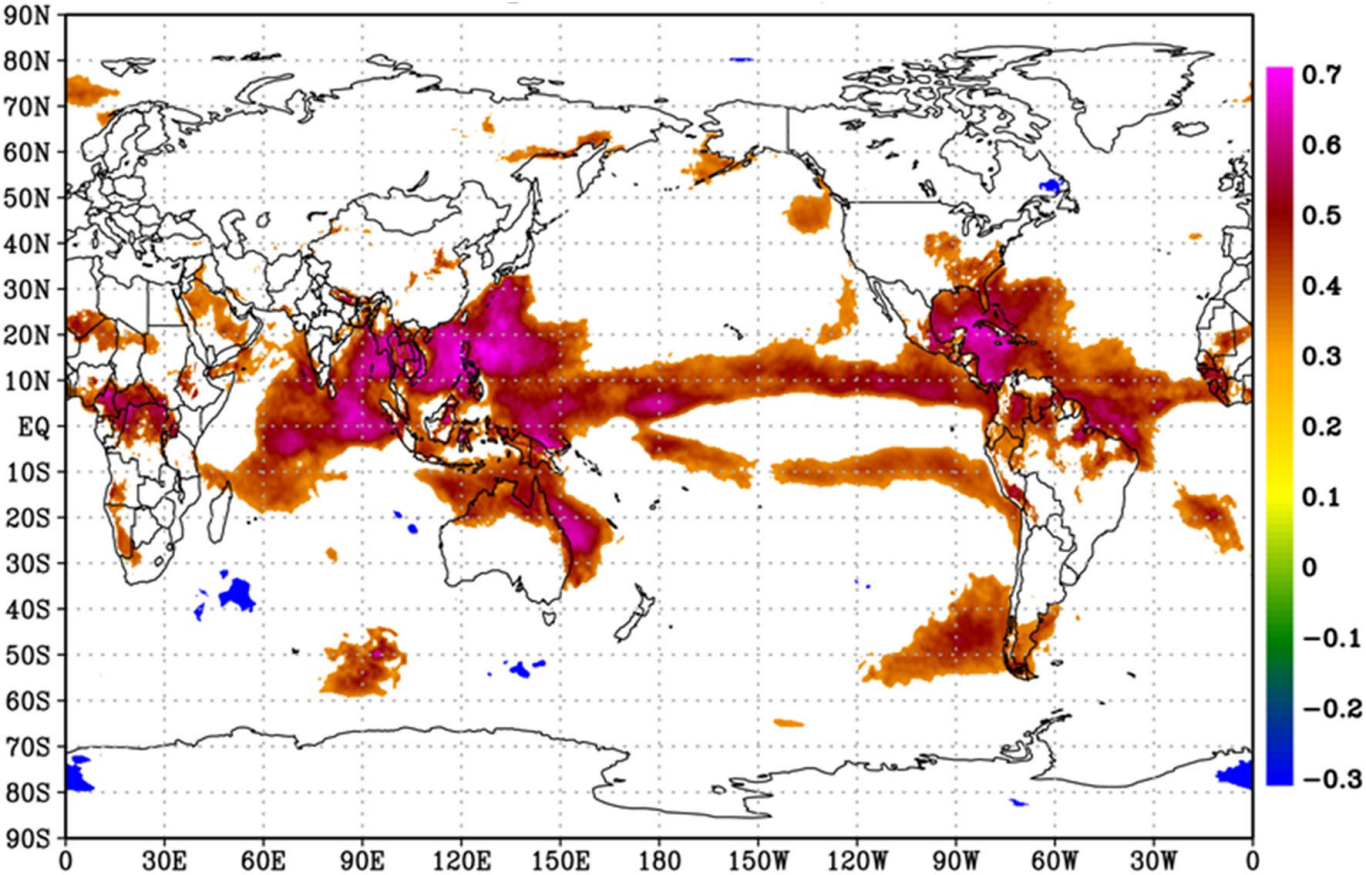
Correlation between area averaged surface pressure over the Tibetan Plateau (TPsfc, 29°N–35°N, 80°E–95°E) and near-surface air temperature at each grid point around the globe. Only correlations that are statistically significant with a 95% confidence interval are displayed. The entire analysis is for June–September, during 1979–2020. The figure is developed using open source software GrADS (Grid Analysis and Display System) version 2.2.1 (http://cola.gmu.edu/grads/downloads.php).

The surface temperature time series correlation with the TPsfc is calculated using 42 years of JJAS data (1979–2020) to investigate the spatial prevalence of the link between TPsfc and surface temperature. The TPsfc is highly positively associated with surface temperature over the tropical zone, with pockets of significant correlations observed all around the globe. Furthermore, over the Bay of Bengal and the tropical western Pacific Ocean, a substantially greater contiguous area of significant correlation (r > 0.60, p < 0.01) was detected (Fig. 6). Surprisingly, the connections over the Tibetan plateau are not significant, with the exception of a significant positive association seen to the south of Tibet (e.g. Nepal). This suggests that local warming plays a minor influence in rising surface pressure over the Tibetan plateau, with regional and hemisphere warming controlling the TPsfc more effectively. It is worth mentioning that there is a strong positive correlation between surface pressure and temperature in the Tibetan region throughout the winter. As summer approaches, the link between these two weakens, although it remains important until April. In a rigorous investigation of the correlation between surface temperature and pressure over the Tibetan Plateau, earlier study also found a strong positive relationship between surface temperature and pressure in the winter season as opposed to the summer season^42^.

We also estimated the relationship between Tibetan surface pressure (TPsfc) and global (GTsfc: 90°S–90°N, 0–360°E) and hemispheric (NHTsfc: 0–90°N, 0–360°E, SHTsfc: 90°S–0, and 0–360°E) averaged surface temperature. The TPsfc had a significant positive relationship with GTsfc (r = 0.29, p < 0.1) and NHTsfc (r = 0.38, p < 0.05), but a non-significant relationship with SHTsfc (r = 0.10). Surface temperature averaged within 0–45°N latitudes (NH45Tsfc; 0°–45°N, 0–360°E) had the strongest connection with TPsfc (r = 0.69, p < 0.01). Furthermore, there are positive trends in the NH45Tsfc (0.23 K decade^−1^, p < 0.01). These warming trends in surface temperature, which are similar to TPsfc trends (0.23 hPa decade^−1^), back up past findings of Tibetan surface pressure sensitivity to regional and hemispheric surface temperature changes. Surface pressure over the Tibetan Plateau and averaged hemispheric surface temperature fields were also found to have a substantial positive association in earlier studies^41,42,68^.

**Figure 7:**
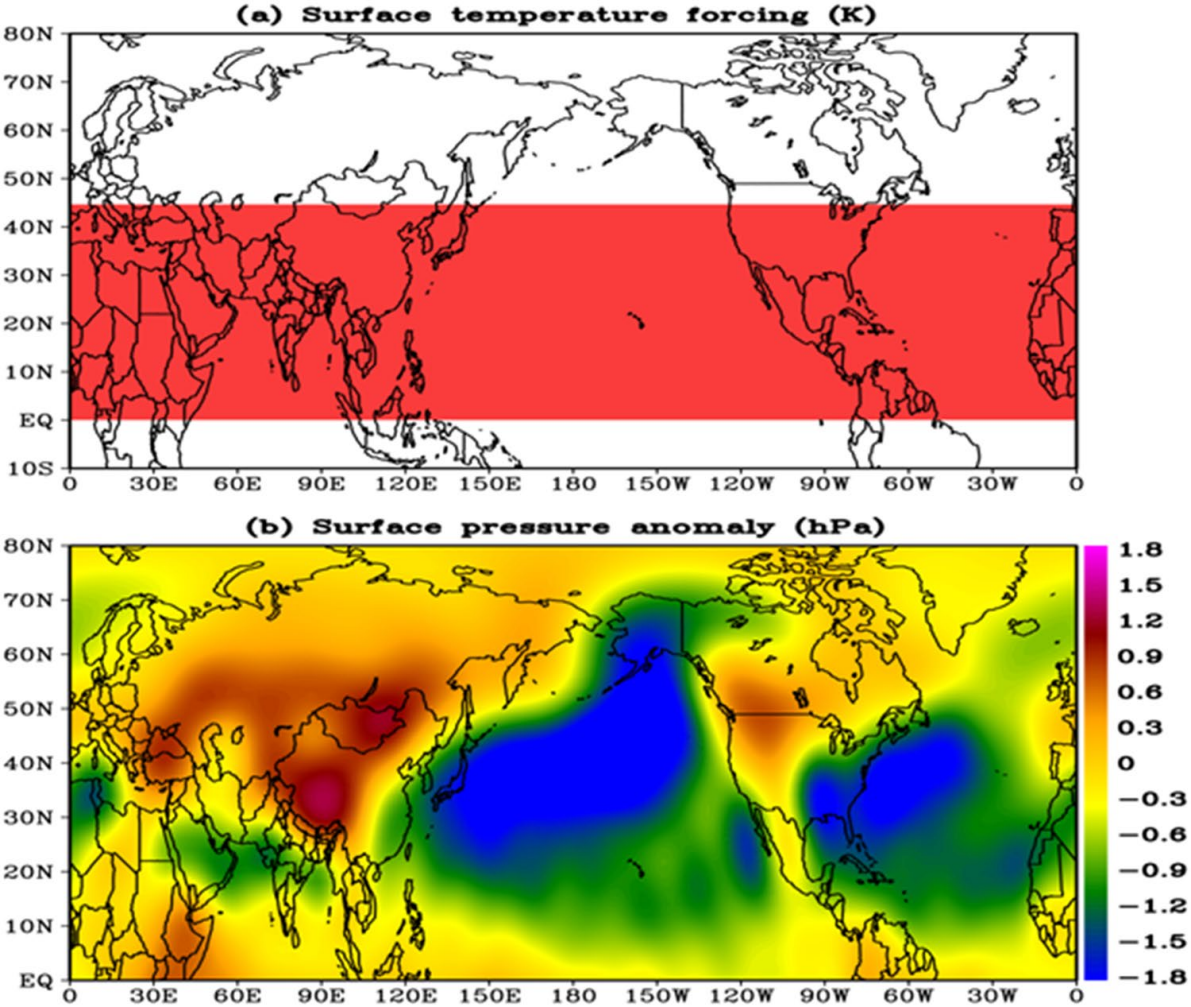
(**a**) Region of warming in the linear baroclinic model, a warming of 1 K was specified at the surface, and in the vertical direction, gamma distribution was used with maximum warming (1 K) at the surface, (**b**) Response of the surface pressure anomalies to the warming. The LBM model was integrated for entire JJAS (June through September), using NCEP reanalysis derived climatology (1958–1997) as the basic atmospheric state. The figures are developed using open source software GrADS (Grid Analysis and Display System) version 2.2.1 (http://cola.gmu.edu/grads/downloads.php).

We also use LBM (Linear Baroclinic Model) to look at how the Tibetan surface pressure responds to hemispheric warming^70^. Two experiments were carried out. The temperature was raised uniformly in the horizontal direction in the first experiment, while in the vertical direction; the gamma distribution was employed with maximum warming (1 K) at the surface. The warming is defined as extending from 0 to 360°E longitude and 0 to 45°N latitude (Fig. 7a). This region was chosen because observational results show that surface temperature averaged over this region has a significant connection (r = 0.69, p < 0.01) with TPsfc. The second experiment is identical to the first, except that the temperature decreased rather than increased. Using NCEP climatology as the baseline state in each experiment, the model was integrated for the entire summer (June through September). Throughout the simulation, the temperature forcing was kept constant. Two positive anomalies across the Tibetan and Mongolian Plateaus (extending up to the Mediterranean region) and over the west coast of America, and two negative anomalies over the Pacific and Atlantic, clearly reveal a wave pattern (Fig. 7b). Over the east coast of South Africa, another band of abnormal positive anomalies may be spotted (e.g. Somalia, Ethiopia, and Kenya). Surprisingly, positive anomaly sites tend to be found in mountainous regions. Surface pressure responses to cooling also exhibit a distinct wave pattern, but the anomalies’ signs have been reversed (not shown for brevity). These findings show that the warming climate on both the regional and hemispheric scales is causing Tibetan surface pressure to rise.

Note that our additional LBL experiments using surface temperature anomalies only over the Bay of Bengal and the tropical western Pacific produced positive but weak surface pressure anomalies over the Tibetan Plateau, despite the strongest correlation between TPsfc and surface temperature over the Bay of Bengal and the tropical western Pacific. This could be due to the fact that LBL only depicts the linear response of the perturbation, not the full atmospheric processes. In contrast, when a horizontal homogeneous temperature anomaly is imposed, the pressure anomaly reveals that warming play a significant role in rising surface pressure across the Tibetan Plateau.

## Discussion

Rainfall in northwest India has been trending upwards, while it has been trending downwards in the Indo-Gangetic plains and northeast India. Despite the fact that this dipole pattern in rainfall trends has been found in numerous previous research, the exact reason for the asymmetry in rainfall trends remained unclear. We show that the dipole pattern in rainfall trends is linked to changes in surface pressure across the Tibetan Plateau. Rainfall increases over northwest India and decreases over the Indo-Gangetic Plains and northeast India as surface pressure over the Tibetan Plateau rises. It’s worth noting that, unlike northwest India, the Indo-Gangetic plains and northeast India’s rainfall regression with Tibetan surface pressure are not uniform. Instead, only a few isolated spots show a substantial correlation, implying that local or remote forcings, in addition to Tibetan surface pressure, may play an important role.

According to the study, surface pressure above the Tibetan Plateau controls the Indian rainfall pattern by regulating both low and middle-level circulation. As a result of rising surface pressure over the Tibetan Plateau, lower level easterlies have intensified in northwestern India, while middle tropospheric north westerlies have decreased across East India. As a result of the above-mentioned changes in the circulation pattern, more water vapour is carried over northwest India. Increased lower-level easterlies, for example, carried more water vapour from the Bay of Bengal to northwest India, while decreased mid-level north westerlies kept extratropical dry air out of India, thereby strengthening the rainfall generation mechanism. The vorticity, which is a key factor that governs convection and rainfall activity in the monsoon trough, is further strengthened by increased easterly over northwest India. Furthermore, while the TPsfc was high, a mid-tropospheric cyclonic circulation anomaly over the central Arabian Sea transported more water vapour from the Arabian Sea to northwest India, resulting in greater rainfall. The cyclonic circulation over East India was weaker due to the high surface pressure over Tibet, resulting in less rainfall in the IGP region.

Granger causality studies show a causative relationship between Tibetan surface pressure and rainfall in northwest India, with a delay of 1 to 13 days.

The surface pressure above the Tibetan Plateau increased significantly between 1979 and 2020. According to both observational and modeling studies, regional/hemispheric warming causes higher surface pressure over the Tibetan Plateau in a hydrostatic atmosphere. Lower level easterlies over northwestern India and middle-level southerlies over East India show a rising tendency from 1979 through 2020, which is consistent with surface pressure patterns over the Tibetan Plateau. Furthermore, the Tibetan Plateau’s enhanced water vapour loading contributes to the surface pressure trends (14%). Furthermore, the results show that the relationship between TPsfc and ISMR is unaffected by ISMR’s interactions with other important climatic factors (e.g. ENSO, PDO, NP, NAO, AO etc.).

It is worth mentioning that, in addition to the increased surface pressure over Tibet, other causes are also contributing to the increased rainfall activity in northwest India. For example, according to a recent study, a rise in surface temperature as well as an intensification of the surface low-pressure area over Iran and anomalous cyclonic circulation over the Northern Arabian Sea is responsible for an increase in rainfall activity in northwest India^16^. The author further said that the surface temperature over Iran has been rising in recent decades. As a result, the south-west monsoon has shifted westward (for example, prolonged north-westward intrusion of low pressure systems (LPSs) form over the Bay of Bengal's head), bringing increased flooding in northwest India and drought in northeast India.

Furthermore, the monsoon low-level jet (MLLJ), a strong cross-equatorial flow in the lower troposphere that originates from Mascarene High and flows across the east coast of Somalia to the Indian peninsula, is critical to the Indian summer monsoon rainfall. Rainfall in northwest India (NWIR) is significantly correlated with winds across MLLJ's core region (5°N–15°N, 50°E–70°E) over the Arabian Sea, according to our analysis (Fig. S12), which shows a correlation of 0.62 (Pvalue < 0.01). Interestingly, the wind intensity in MLLJ has been increasing over the last four decades (0.22 ms^−1^decade^−1^, Pvalue < 0.01). Using data from 1950 to 2015, earlier study also reported a rise in low-level monsoon westerlies across the Arabian Sea^24^. Since strong cross-equatorial flow (i.e. MLLJ) is a manifestation of large thermal gradients between the Asian landmass and surrounding oceans, the intensification of MLLJ could be attributed to rising temperatures over Iran.

Similarly, there is evidence that human activities are influencing rainfall in the IGP region^71^. Anthropogenic activities such as increased greenhouse gas emissions, changes in radiative forcing due to aerosols and clouds, and changes in land surface physical properties due to land use changes such as urbanization and agricultural practices are some of the anthropogenic activities that may be playing a role, in addition to TPsfc changes. The IGP, India's largest irrigated region, is home to 40% of the country's population and 50% of its irrigated land, with groundwater irrigation serving as the primary source of water. Irrigated agriculture in India has expanded from less than 20% in the 1960s to more than 45% today. Increased irrigation, according to a recent modeling study, decreased the monsoon circulation, resulting in less precipitation^72^. As a result, these various forcings may interact, leading to a complex change in monsoon rainfall patterns.

Our findings are important in determining what is causing regional variations in the Indian summer monsoon rainfall pattern. The findings of this study could aid in better understanding and forecasting summer monsoon rainfall on interannual time scales. The findings will be useful in hydrological planning for the country under the backdrop of global warming because the surface pressure anomalies over the Tibetan Plateau are extremely sensitive to regional/hemispheric warming. Furthermore, the significant synoptic-scale link between Tibetan surface pressure and ISMR can help with long-range monsoon forecasting.

## Methods

### Data sets

The present study utilizes three datasets that are listed below.

1. We use the daily Indian summer monsoon (June through September, JJAS) precipitation data set obtained from rain-gauge observations by India Meteorological Department (IMD)^73^, with 0.25° × 0.25° spatial resolution, for the period 1979–2020. The seasonal mean (JJAS) rainfall was obtained by accumulating the daily average precipitation.
2. Surface pressure, surface temperature (i.e. 2-m height), total precipitable water vapour, specific humidity and winds (zonal, meridional, and vertical) at multiple levels (850, 500, 300 and 200 hPa) are extracted from ERA5 reanalysis, for the period 1979–2020. The temporal resolution of ERA5 data used in the present study varies from daily to monthly. ERA5 is the fifth generation atmospheric reanalysis of the global climate covering the period from January 1950 to the present from the European Centre for Medium-Range Weather Forecasts^74^. The data cover the Earth with 0.25° × 0.25° spatial resolution and resolve the atmosphere using 137 levels (1000 hPa to 1 hPa) from the surface up to a height of 80 km.
3. The various monthly climate indices, North Atlantic Oscillation (NAO) index, Arctic Oscillation (AO), Nino 3.4 index (https://psl.noaa.gov/data/climateindices/list), Pacific Decadal Oscillation (PDO, http://jisao.washington.edu/pdo), North Pacific Pattern (NP, https://climatedataguide.ucar.edu ), have also been used from 1979 to 2020.

### Data analysis

Composites, correlation, and regression analysis were used to assess the relationship between Tibetan surface pressure and Indian summer monsoon rainfall (ISMR). To confine to interannual variability, all-time series were detrended before the regression analysis. To investigate the relationship between Tibetan surface pressure and ISMR, a Granger causality analysis was used. The correlation coefficient’s significance was determined using a standard two-tailed Student’s t-test. The LBM version 2.3 was used to run sensitivity tests in order to better understand how warming affects the surface pressure over the Tibetan Plateau.

## Supplementary Information

Supplementary Figures.

## Data Availability

All datasets used in this study are publicly available. The ERA5 data are retrieved from https://cds.climate.copernicus.edu. The IMD rainfall data were taken from https://imdpune.gov.in. The various climatic indices were obtained from https://psl.noaa.gov/data/climateindices/list, http://jisao.washington.edu/pdo and https://climatedataguide.ucar.edu.

## Code availability

The LBM used in this study is obtained from https://ccsr.aori.u-tokyo.ac.jp/~lbm/sub/lbm.html.
