# Supplementary material for: Rising surface pressure over Tibetan Plateau strengthens indian summer monsoon rainfall over northwestern India
Source: Sci Rep. 2022 May 21;12:8621. doi: 10.1038/s41598-022-12523-8 (PMC9124204; doi:10.1038/s41598-022-12523-8)
Supplement: Supplementary file 1 — Supplementary Figures. [file 41598_2022_12523_MOESM1_ESM.pdf]

Supplementary Information

Rising Surface Pressure over Tibetan Plateau Strengthens Indian Summer Monsoon Rainfall over Northwestern India

[*]Randhir Singh[1], Neeru Jaiswal[1] and C M Kishtawal[1]

[1]Space Applications Centre, Indian Space Research Organisation (ISRO), Ahmedabad-380015, India

[*]Corresponding Author Address:
randhir_h@yahoo.com

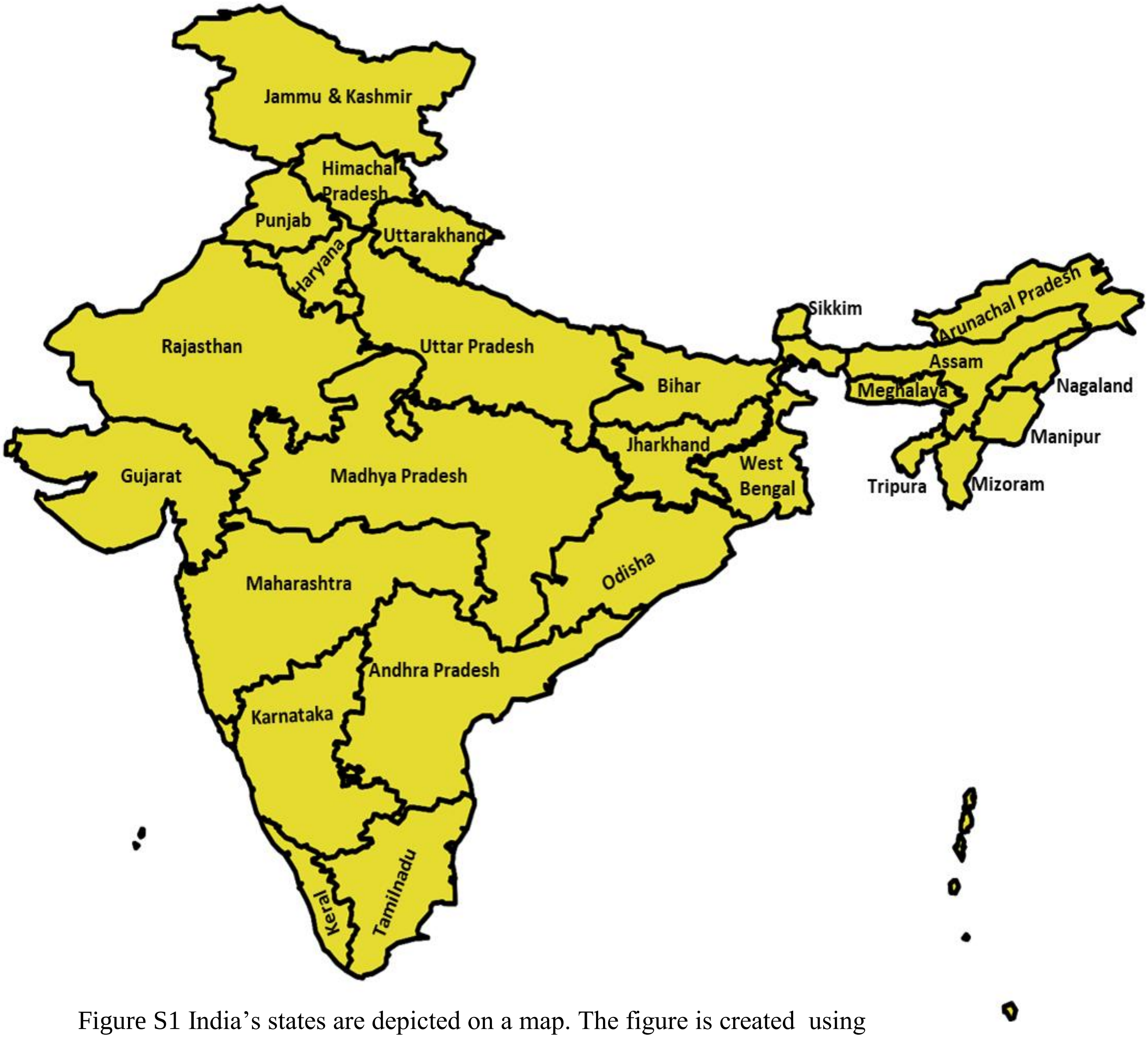

Figure S1 India's states are depicted on a map. The figure is created using open source software GrADS (Grid Analysis and Display System) version 2.2.1 (http://cola.gmu.edu/grads/downloads.php).

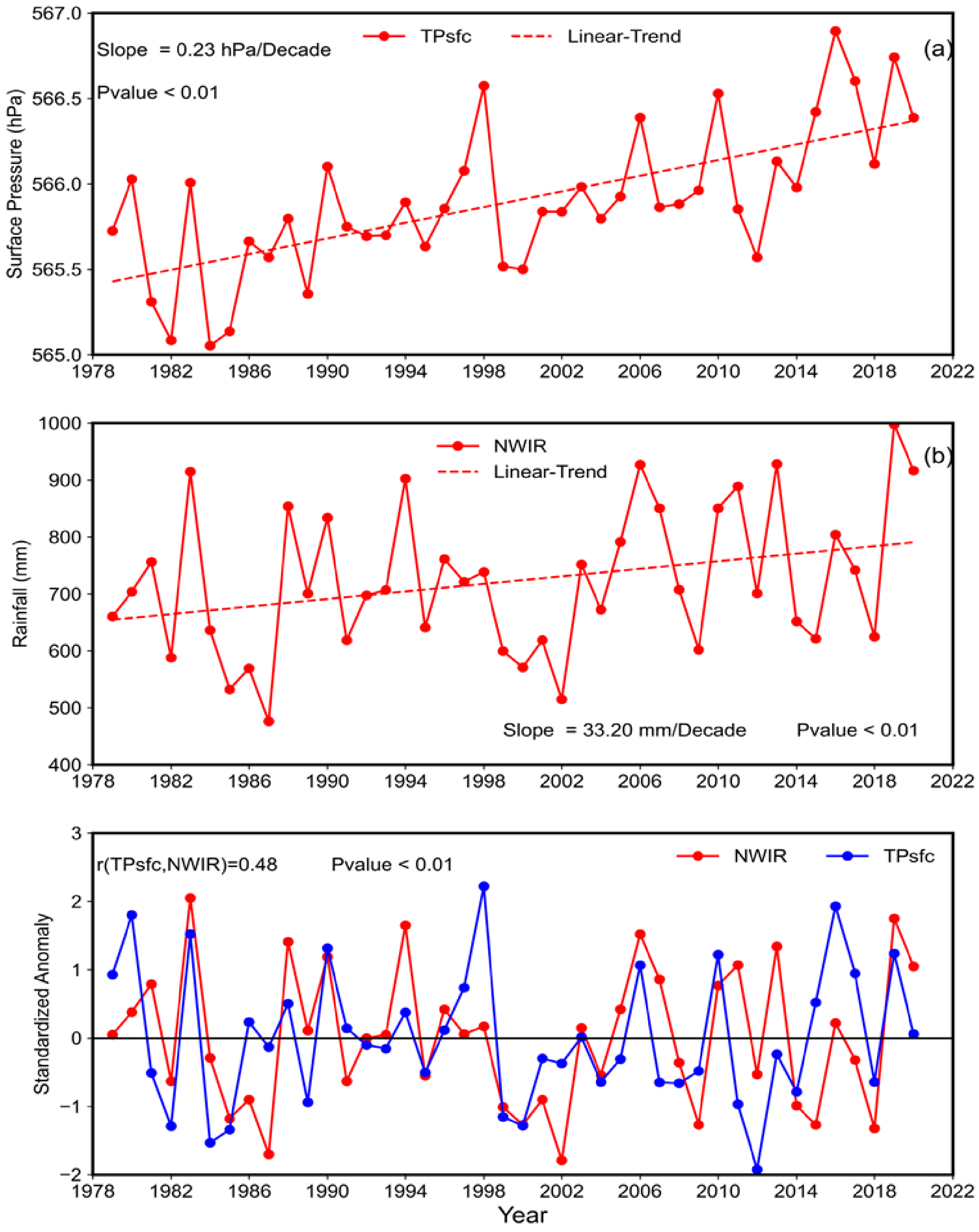

Figure S2 Time series of area averaged (TPsfc: 29°N-35°N, 80°E-95°E) surface pressure over the Tibetan Plateau (a), area averaged (NWIR: 15°N-28°N, 68°E-78°E) rainfall over north west India (b), and detrended and standardized time series of TPsfc and NWIR (c). The entire analysis is for June-September, during 1979-2020. The figures are created with Python version 3.9.1 (https://www.python.org), an open source programming language.

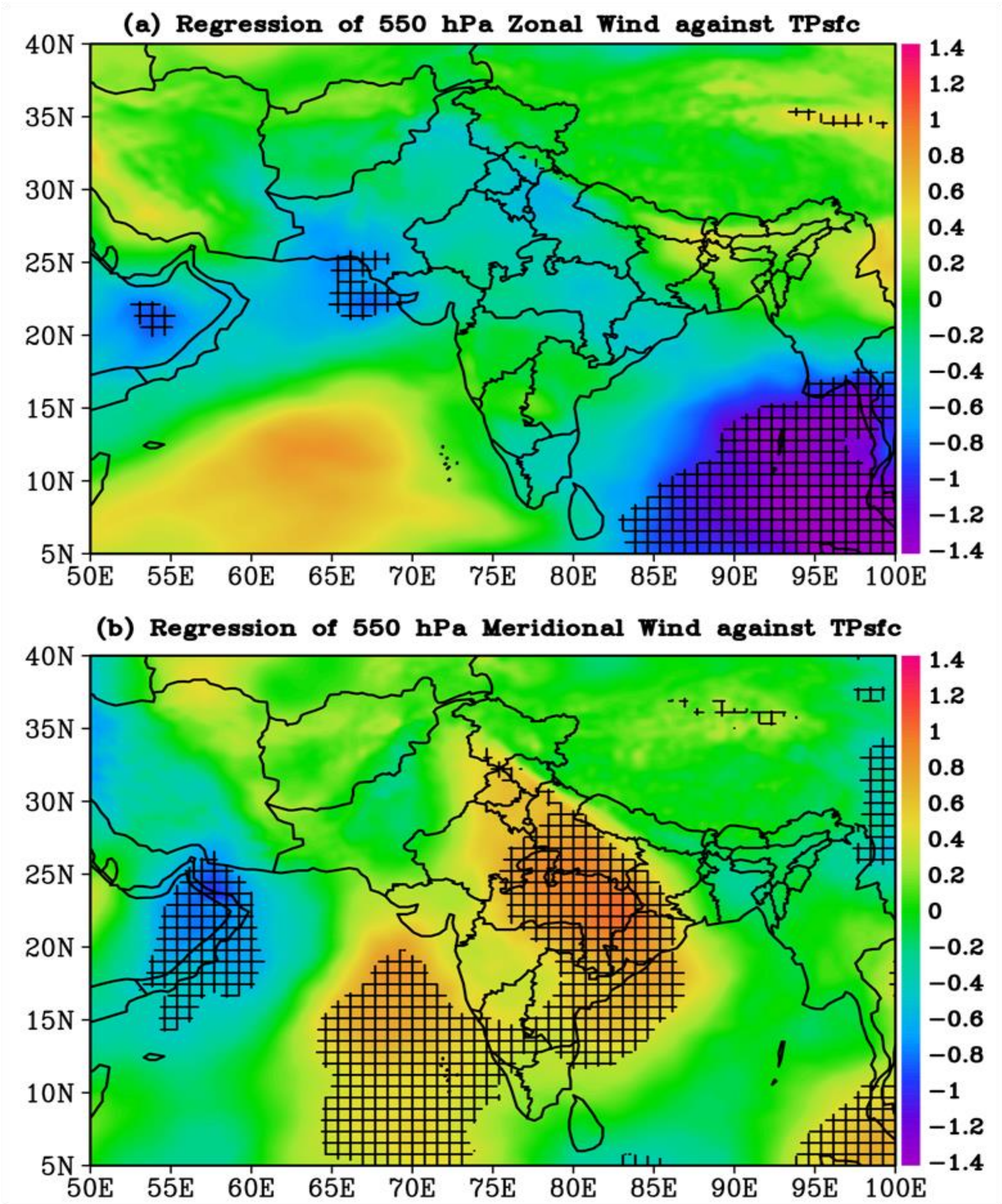

Figure S3 Regression of 550 hPa wind against TPsfc ($ms^{-1}hPa^{-1}$), (a) for zonal wind, and (b) for meridional wind. The black hatch area in indicates the regions where regressions are significant above 95% confidence level. The entire analysis is for June-September, during 1979-2020. The figures are created using open source software GrADS (Grid Analysis and Display System) version 2.2.1 (http://cola.gmu.edu/grads/downloads.php).

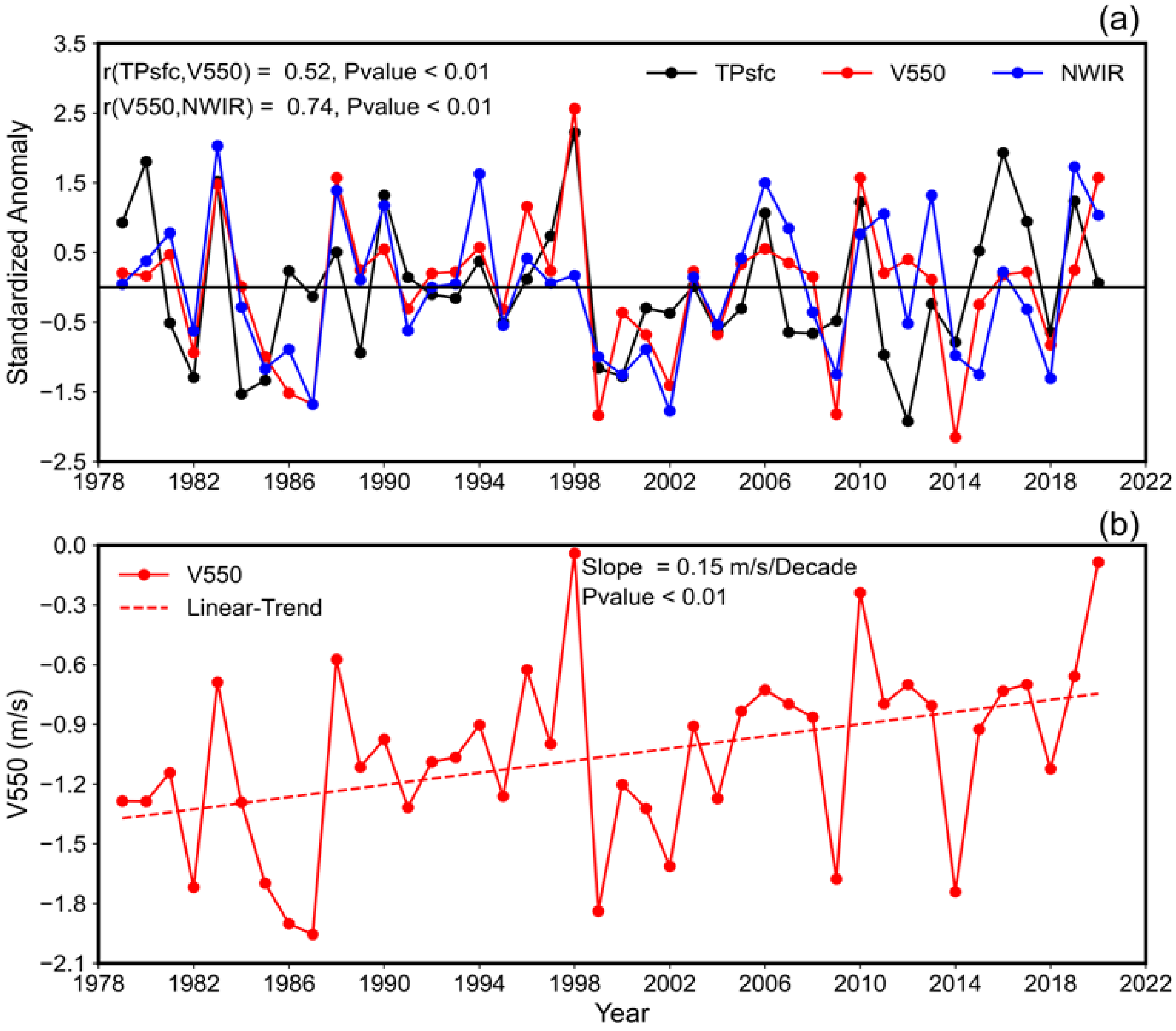

Figure S4 (a) Detrended and standardized time series of area averaged surface pressure over the Tibetan Plateau (TPsfc, 29°N-35°N, 80°E-95°E), 550 hPa meridional wind wind (V550, 20°N-30°N, 68°E-78°E), area averaged rainfall (NWIR, 15°N-28°N, 75°E-85°E), and (b) time series of 550 hPa meridional wind (V550, 15°N-28°N, 75°E-85°E). The entire analysis is for June-September, during 1979-2020. The figures are created with Python version 3.9.1 (https://www.python.org), an open source programming language.

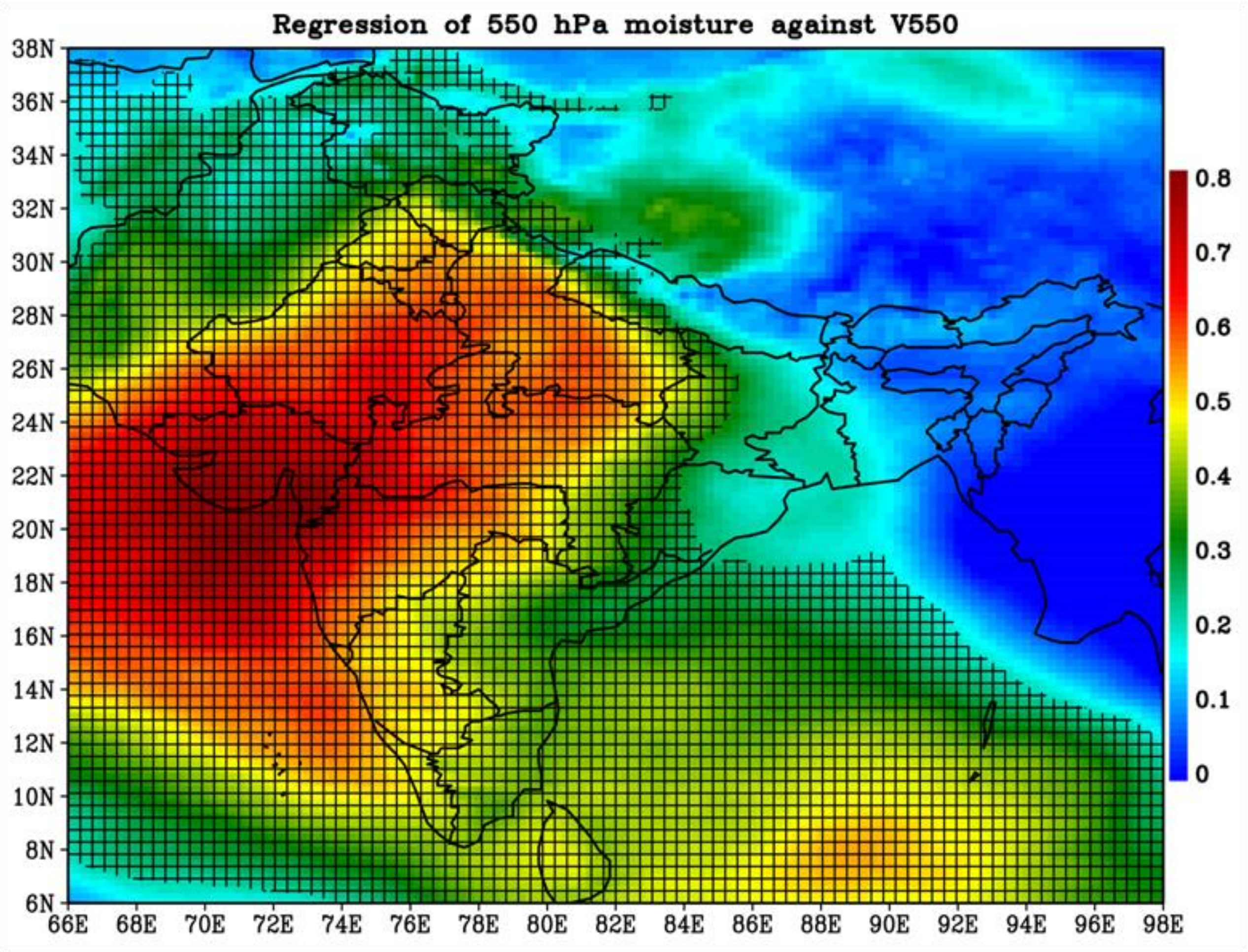

Figure S5 Regression of 550 hPa specific humidty against V550 (gkg^{-1}/ms^{-1}), The black hatch area in indicates the regions where regressions are significant above 95% confidence level. The entire analysis is for June-September, during 1979-2020. The figure is created using open source software GrADS (Grid Analysis and Display System) version 2.2.1 (http://cola.gmu.edu/grads/downloads.php).

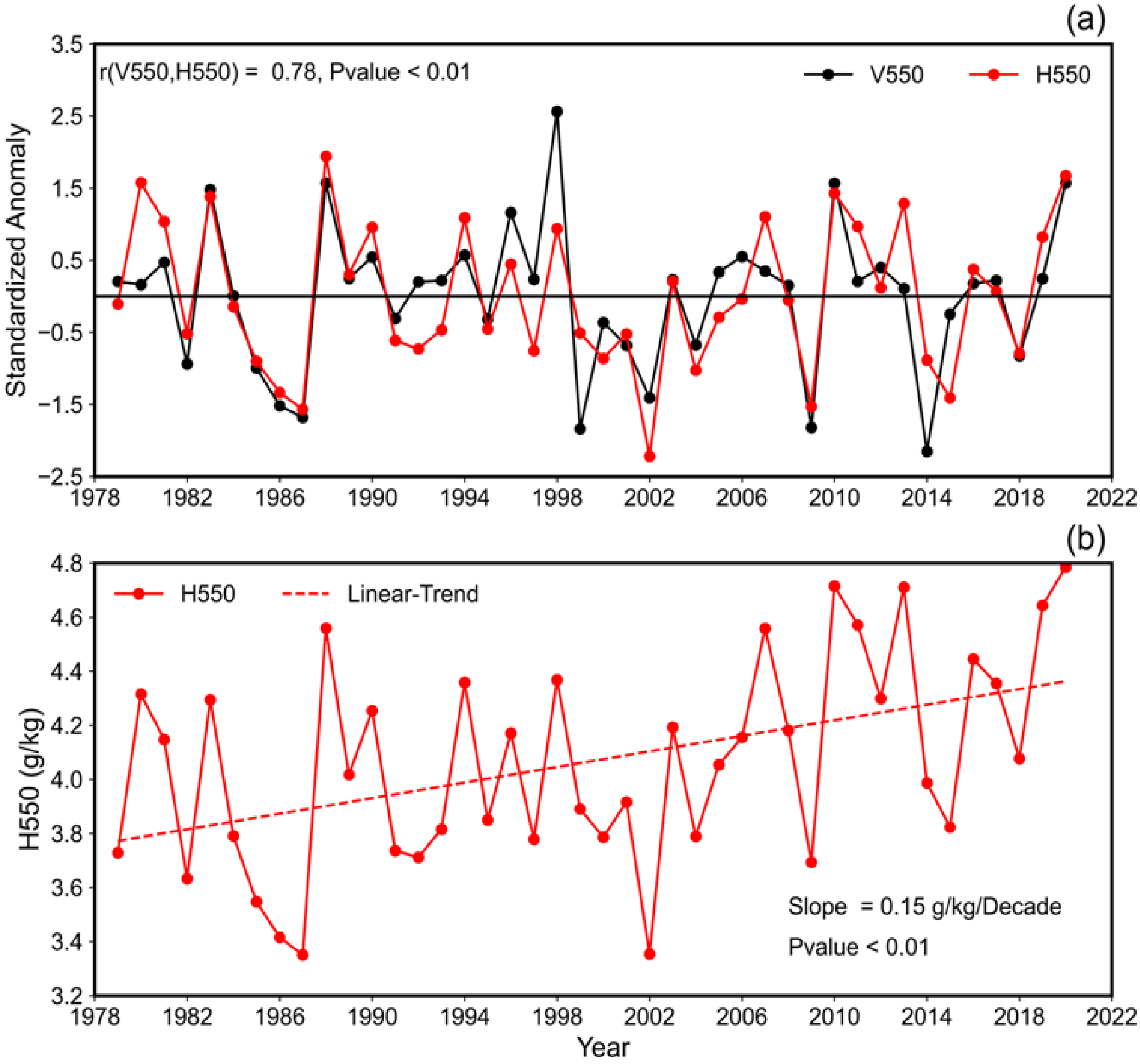

Figure S6 (a) Detrended and standardized time series of area averaged 550 hPa meridional wind (V550, 15°N-28°N, 75°E-85°E) and area averaged 550 hPa specific humidity (H550, 15°N-28°N, 68°E-78°E), and (b) time series of 550 hPa specific humidity (H550, 15°N-28°N, 68°E-78°E). The entire analysis is for June-September, during 1979-2020. The figures are created with Python version 3.9.1 (https://www.python.org), an open source programming language.

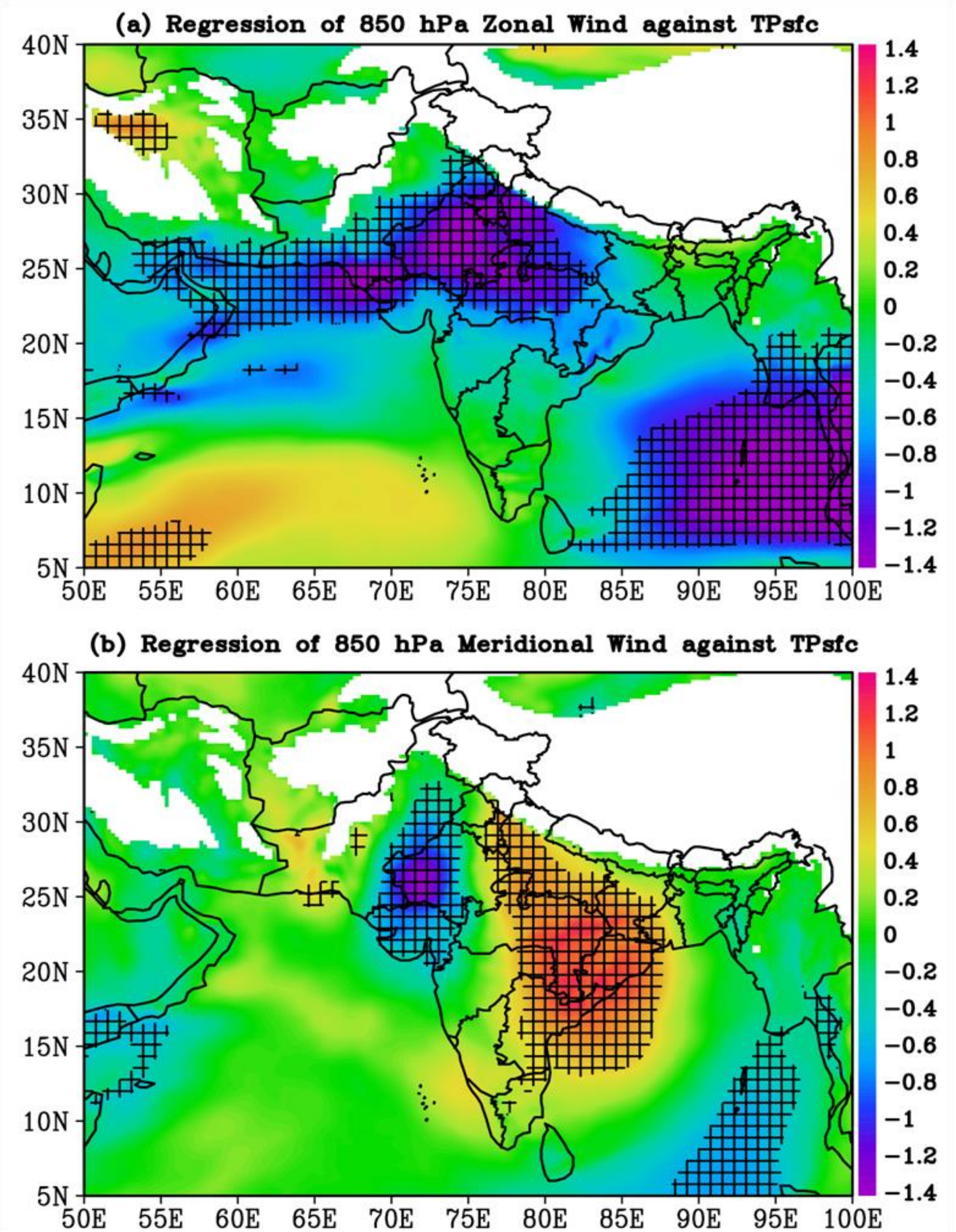

Figure S7 Regression of 850 hPa wind against TPsfc ($ms^{-1}hPa^{-1}$), (a) for zonal wind, and (b) for meridional wind. The black hatch area in indicates the regions where regressions are significant above 95% confidence level. The entire analysis is for June-September, during 1979-2020. The figures are created using open source software GrADS (Grid Analysis and Display System) version 2.2.1 (http://cola.gmu.edu/grads/downloads.php).

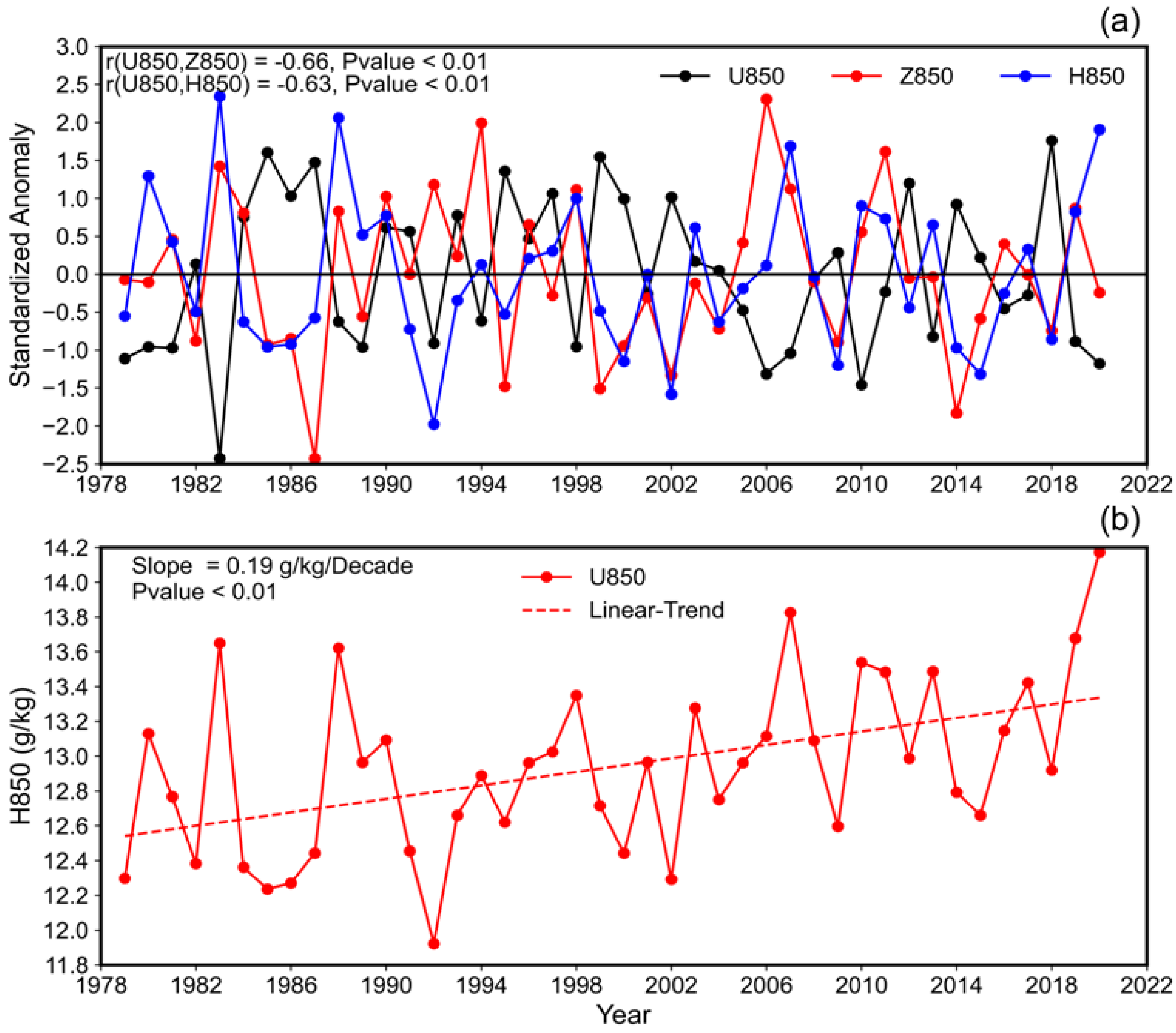

Figure S8 (a) Detrended and standardized time series of area averaged 850 hPa zonal wind (U850, 22°N-30°N, 68°E-78°E), area averaged 850 hPa specific humidity (H850, 15°N-28°N, 68°E-78°E), area averaged 850 hPa relative vorticity (Z850, 15°N-28°N, 70°E-78°E) and (b) time series of 850 hPa specific humidity (H550, 15°N-28°N, 68°E-78°E). The entire analysis is for June-September, during 1979-2020. The figures are created with Python version 3.9.1 (https://www.python.org), an open source programming language.

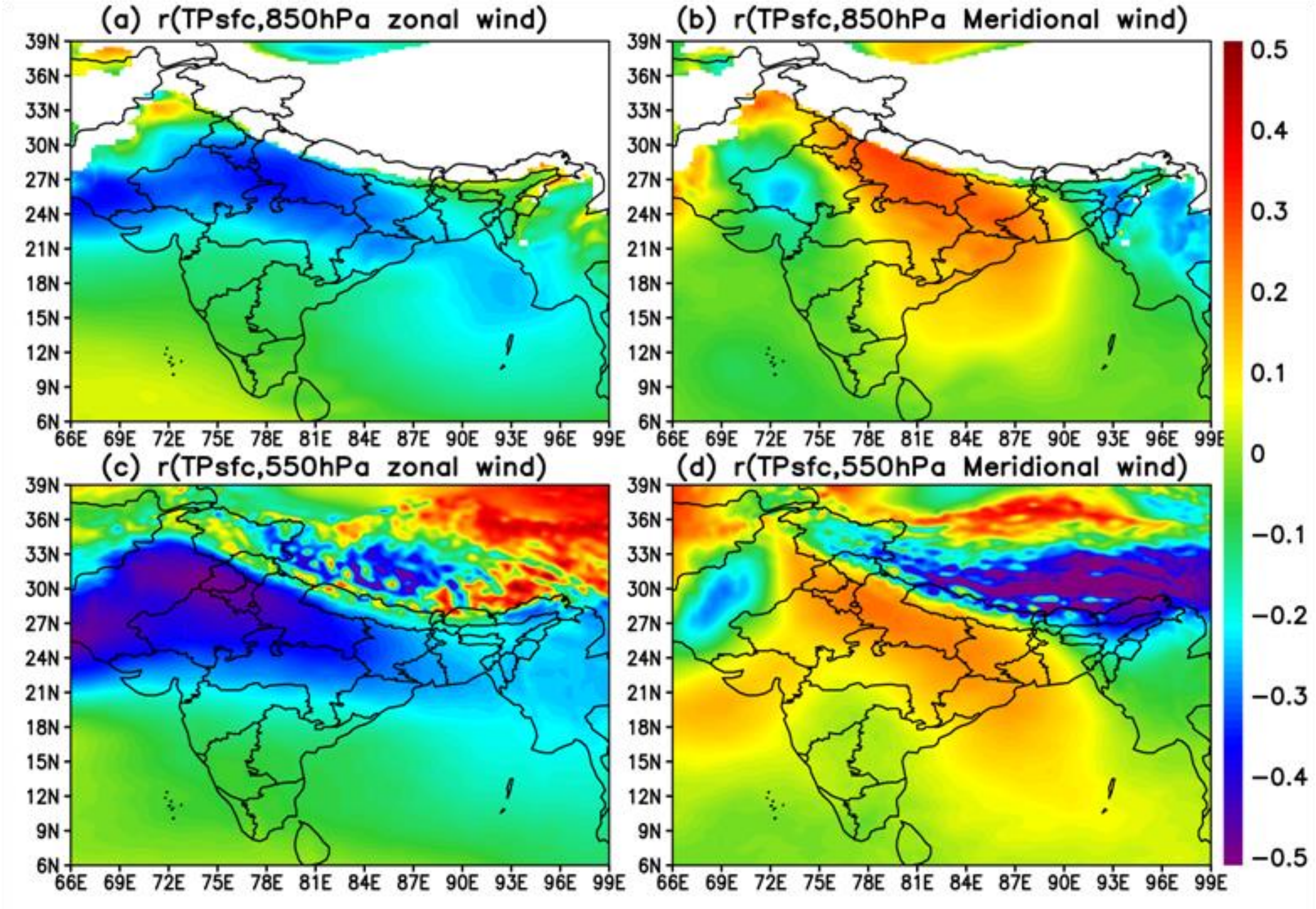

Figure S9 Correlation of area averaged surface pressure over the Tibetan Plateau (TPsfc, 29°N-35°N, 80°E-95°E) with, (a) 850 zonal wind, (b) 850 hPa meridional wind , (c) 550 zonal wind, and (d) 550 hPa meridional wind. The entire analysis is based on daily averaged surface pressure and winds for the months of June to September from 1979 to 2020. A correlation |r| greater than 0.04 is statistically significant at a 99 percent confidence level. The figure are created using open source software GrADS (Grid Analysis and Display System) version 2.2.1 (http://cola.gmu.edu/grads/downloads.php).

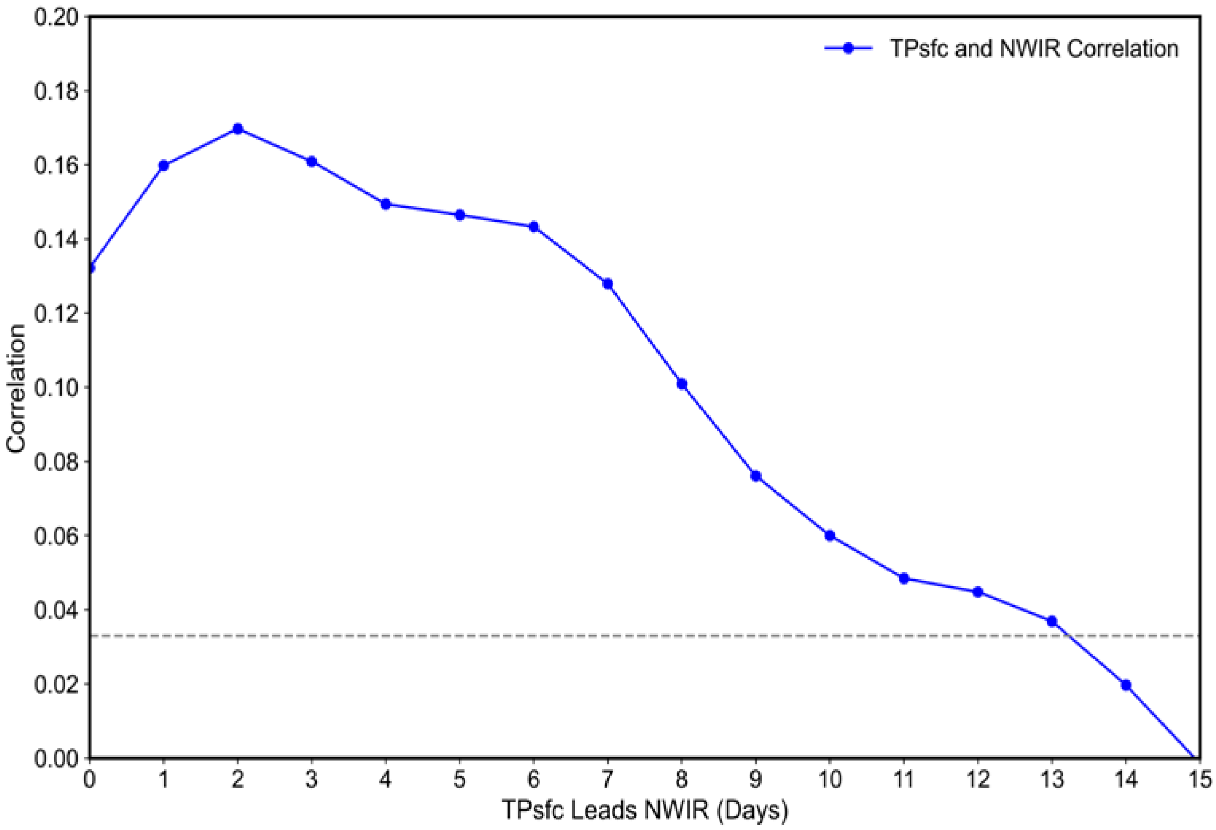

Figure S10 Lead-lag correlation of TPsfc with respect to rainfall (i.e. NWIR), computed using daily surface pressure and rainfall data. The dotted horizontal line represents the 95% confidence level. The analysis is for June-September, during 1979-2020. The figure is created with Python version 3.9.1 (https://www.python.org), an open source programming language.

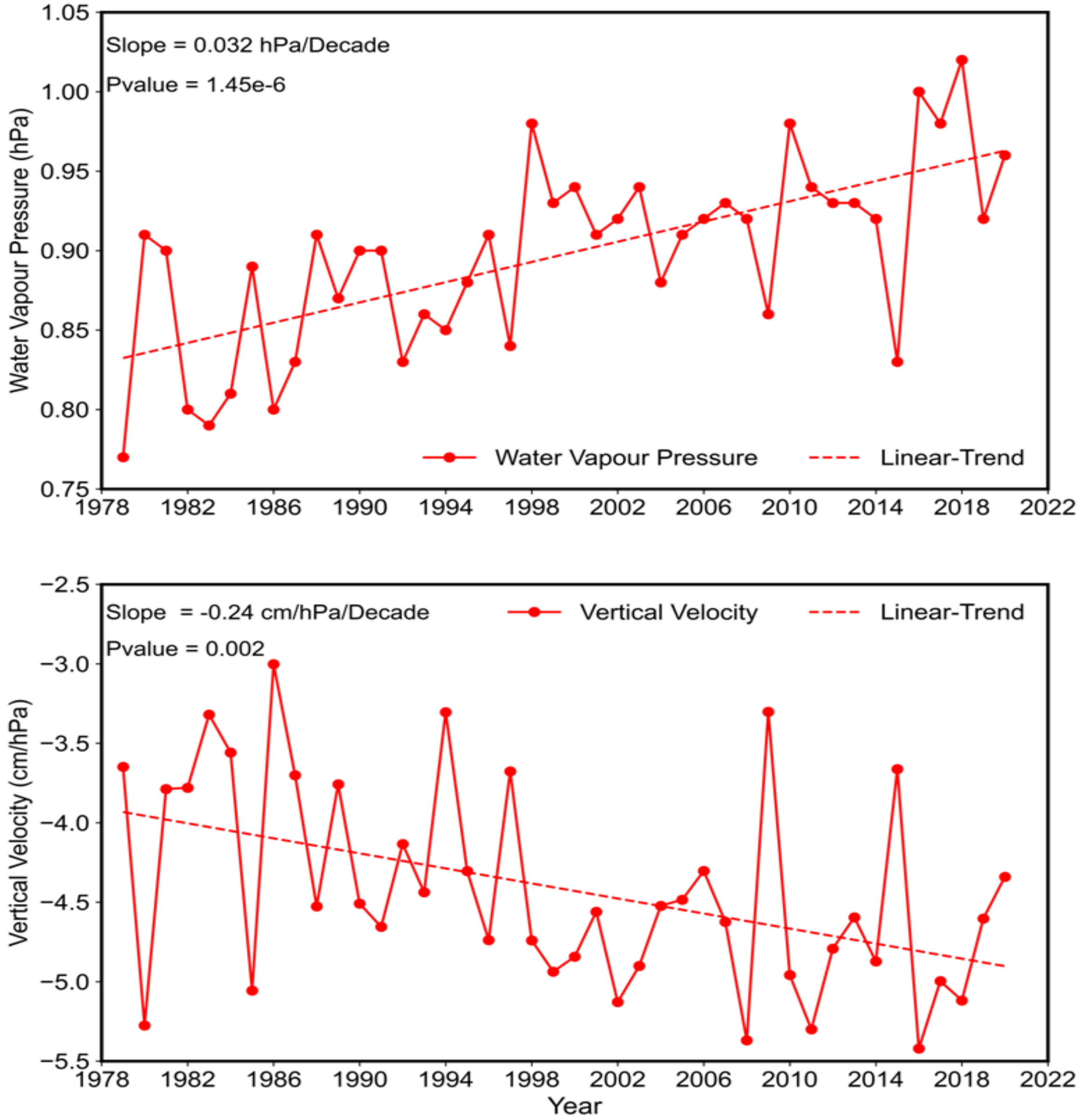

Figure S11 Time series of water vapour pressure (a) and 300 hPa vertical velocity (b) over the Tibetan Plateau (29°N-35°N, 80°E-95°E). The entire analysis is for June-September, during 1979-2020. Note that negative values of vertical velocity indicate upward motion. The figures are created with Python version 3.9.1 (https://www.python.org), an open source programming language.

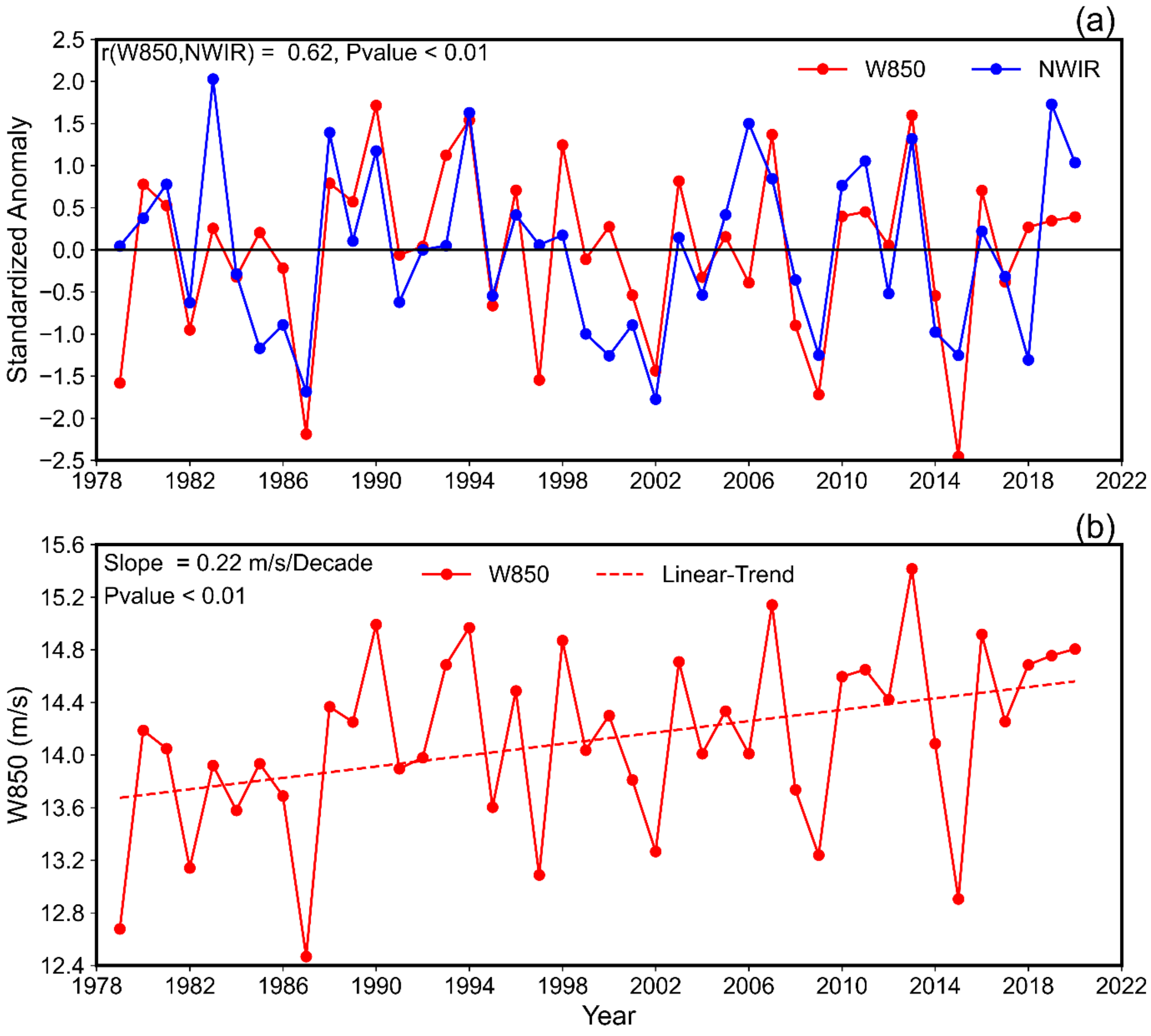

Figure S12 (a) Detrended and standardized time series of area averaged 850 hPa wind speed (W850, 5°N-15°N, 50°E-70°E), area averaged (NWIR: 15°N-28°N, 68°E-78°E) rainfall over north west India, and (b) time series of 850 hPa wind speed (W850, 5°N-15°N, 50°E-70°E). The entire analysis is for June-September, during 1979-2020. The figures are created with Python version 3.9.1 (https://www.python.org), an open source programming language.
